# Hematopoietic differentiation is characterized by a transient peak of entropy at a single-cell level

**DOI:** 10.1186/s12915-022-01264-9

**Published:** 2022-03-09

**Authors:** Charles Dussiau, Agathe Boussaroque, Mathilde Gaillard, Clotilde Bravetti, Laila Zaroili, Camille Knosp, Chloé Friedrich, Philippe Asquier, Lise Willems, Laurent Quint, Didier Bouscary, Michaela Fontenay, Thibault Espinasse, Adriana Plesa, Pierre Sujobert, Olivier Gandrillon, Olivier Kosmider

**Affiliations:** 1 Institut Cochin, CNRSUMR8104, INSERM U1016, OPALE Carnot Institute, The Organization for Partnerships in Leukemia, Université de Paris, Paris, France; 2 Assistance Publique-Hôpitaux de Paris, Centre - Université de Paris, Service d’Hématologie biologique, Hôpital Cochin, Paris, France; 3 ENS de Lyon, Université Claude Bernard, CNRS UMR 5239, INSERM U1210, Laboratory of Biology and Modelling of the Cell, Université de Lyon, 46 allée d’Italie Site Jacques Monod, F-69007 Lyon, France; 4 Pole Sante Leonard de Vinci, Chambray Les Tours, France; 5 Assistance Publique-Hôpitaux de Paris, Centre-Université de Paris, Service d’Hématologie Clinique, Hôpital Cochin, Paris, France; 6 Hôpital Américain de Paris, 63 boulevard Victor Hugo, 92200 Neuilly-sur-Seine, France; 7 Université Lyon 1, CNRS UMR5208, Institut Camille Jordan, Université de Lyon, 43 blvd. du 11 novembre 1918, F-69622 Villeurbanne Cedex, France; 8 Inria Team Dracula, Inria Center Grenoble Rhône-Alpes, Montbonnot-Saint-Martin, France; 9 Hospices Civils de Lyon, Hôpital Lyon Sud, Service d’Hématologie Biologique, Pierre-Bénite, France; 10 Cancer Research Center of Lyon, INSERM U1052 UMR CNRS 5286, Lyon, France

**Keywords:** Hematopoiesis, Single-cell RNA-seq, Cell-to-cell variability, Entropy, Myelodysplastic syndromes

## Abstract

**Background:**

Mature blood cells arise from hematopoietic stem cells in the bone marrow by a process of differentiation along one of several different lineage trajectories. This is often represented as a series of discrete steps of increasing progenitor cell commitment to a given lineage, but as for differentiation in general, whether the process is instructive or stochastic remains controversial. Here, we examine this question by analyzing single-cell transcriptomic data from human bone marrow cells, assessing cell-to-cell variability along the trajectories of hematopoietic differentiation into four different types of mature blood cells. The instructive model predicts that cells will be following the same sequence of instructions and that there will be minimal variability of gene expression between them throughout the process, while the stochastic model predicts a role for cell-to-cell variability when lineage commitments are being made.

**Results:**

Applying Shannon entropy to measure cell-to-cell variability among human hematopoietic bone marrow cells at the same stage of differentiation, we observed a transient peak of gene expression variability occurring at characteristic points in all hematopoietic differentiation pathways. Strikingly, the genes whose cell-to-cell variation of expression fluctuated the most over the course of a given differentiation trajectory are pathway-specific genes, whereas genes which showed the greatest variation of mean expression are common to all pathways. Finally, we showed that the level of cell-to-cell variation is increased in the most immature compartment of hematopoiesis in myelodysplastic syndromes.

**Conclusions:**

These data suggest that human hematopoietic differentiation could be better conceptualized as a dynamical stochastic process with a transient stage of cellular indetermination, and strongly support the stochastic view of differentiation. They also highlight the need to consider the role of stochastic gene expression in complex physiological processes and pathologies such as cancers, paving the way for possible noise-based therapies through epigenetic regulation.

**Supplementary Information:**

The online version contains supplementary material available at 10.1186/s12915-022-01264-9.

## Background

Complex biological processes such as development or differentiation are often conceptualized as the execution of a program encoded in the genome. However, the existence of random processes is increasingly recognized, especially at the level of gene expression, as a fundamentally stochastic process, leading to cell-to-cell variations in mRNA and protein levels [1].

Hematopoiesis is a finely regulated process by which hematopoietic stem cells (HSCs) give rise to mature blood cells that belong to myeloid or lymphoid lineages. HSC differentiation toward a lineage is regarded as a continuous process involving a series of increasingly committed progenitors [2] that follow one of several trajectories leading to the production of the various mature blood cells. Whether the process of hematopoietic stem cell commitment is instructive or stochastic has long been the subject of controversies [3, 4]. According to the instructive model, HSCs receive external signals such as cytokines which actively induce them to differentiate into a given lineage [5–7]. On the contrary, the stochastic model proposes that spontaneous stochastic variations of cell phenotype are followed by a selection step driven by cytokines [8, 9]. Following the instructive model, cell-to-cell phenotypic variability should be limited and should not vary during differentiation, since all the cells that committed to a given lineage are following the same instructions. On the contrary, the stochastic model predicts that cell-to-cell variability should increase to reach a maximum level at the stage where the selection occurs.

Until recently, the analysis of hematopoietic differentiation has relied on transcriptomic analyses of sorted cell bulk populations defined by their common phenotype, or on the analysis of only a few markers captured at the single-cell level by flow cytometry. With the technological breakthrough of single-cell transcriptomics [10], we are now able to study each cell’s whole transcriptome during the differentiation process, hence allowing a comprehensive measure of cell-to-cell variability. Based on the quantification of a limited number of mRNAs, we have shown that a surge in gene expression variability occurs during avian erythropoiesis in an in vitro model [11]. This was further confirmed in experimental differentiation models in vitro [12–15] and in animals [16, 17]. Moreover, variability in gene expression has been suggested to play a causal role in cell differentiation leading to the use of single-cell approaches in many studies [15, 18]. However, these observations were limited either by the number of genes that were analyzed [11, 12, 16, 17] or by the question of the physiological relevance of the established cell lines used [14].

Here, we used the conceptual framework of Shannon entropy as a proxy of cell-to-cell variation, to analyze, for each gene, cell-to-cell gene expression variability during HSC differentiation in normal human bone marrow single-cell RNA sequencing (scRNA-seq) datasets. We observed a peak of entropy along all the differentiation pathways (erythroid, granulocytic, dendritic, and B lymphoid). Notably, genes with the highest entropy variation, in a given differentiation pathway, corresponded to genes known as pathway-specific whereas genes with the highest expression variation were common to all pathways. Finally, we analyzed the bone marrow from patients with myelodysplastic syndromes which are characterized by ineffective differentiation; by using the same approach as above, we observed, in the affected patients, a higher level of entropy in the most immature states of differentiation.

## Results

### A peak in cell-to-cell gene expression variability is a common feature of all hematopoietic differentiation lineages

In order to study the stochastic gene expression during normal hematopoiesis, we analyzed public scRNA-seq profiles of 15,962 genes, in 12,602 mononuclear cells derived from the bone marrow of a healthy donor (HBM1) [19, 20]. The expression data were analyzed with Seurat [21], and the cells were individually annotated with SingleR [22] to associate the transcriptome of each cell with the gene expression profile of hematopoietic populations [23]. The Uniform Manifold Approximation and Projection for Dimension Reduction (UMAP) layout of these data distinguished 34 different sub-populations of hematopoietic cells (Additional file 1: Fig. S1A). Interestingly, four differentiation pathways (erythropoiesis, granulopoiesis, dendritic maturation, and B lymphopoiesis), starting from hematopoietic stem cells (CD34+ HSC), were clearly identified (Additional file 1: Fig. S1C). We also showed, for each sub-population, the expression of classical markers [23–26] to validate that these data are representative of normal hematopoiesis (Additional file 1: Fig. S1B).

Using the Slingshot package [27], we first sorted the cells along a pseudotime from the most immature (CD34+ HSC) to the most mature states available in all the differentiation pathways with sufficient cell numbers (Additional file 1: Table S1). We then computed intercellular entropy as a measure of cell-to-cell gene expression variability over a sliding window of 50 cells moving along the pseudotime, with a step of 10 cells (Additional file 1: Fig. S2).

**Fig. 1:**
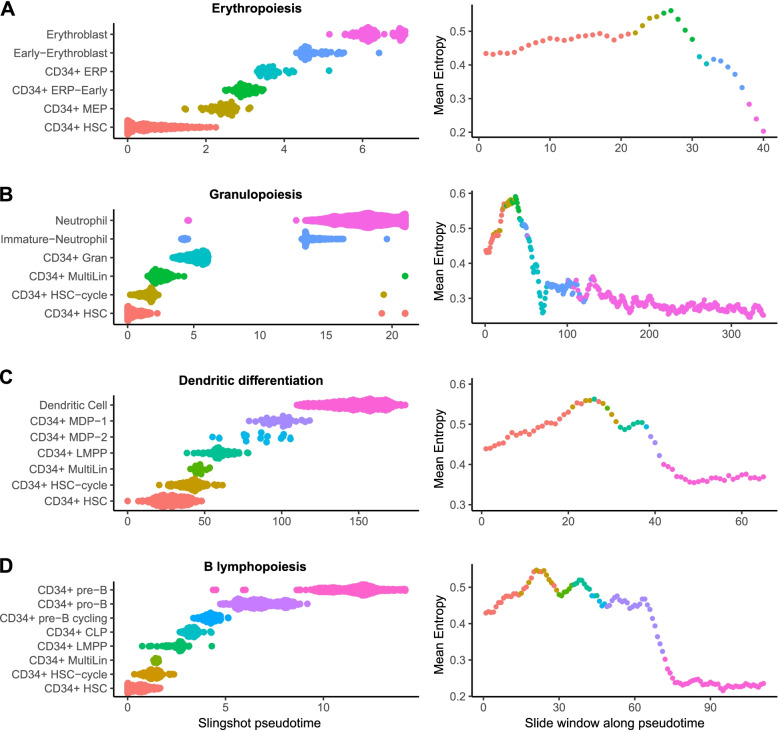
Evolution of cell-to-cell gene expression variability during the main pathways of normal hematopoietic differentiation (HBM1). Cell populations belonging to each differentiation pathway were first selected and then ordered according to the pseudotime calculated by Slingshot. The average intercellular entropy of all genes was then calculated on a sliding window of 50 cells which moves across the pseudotime with a step of 10 cells (the color of each point on the graph correspond to the nature of the first cell in the corresponding sliding window). **A** Erythropoiesis. **B** Granulopoiesis. **C** Dendritic differentiation. **D** B lymphopoiesis

For erythropoiesis, 444 cells were ordered as expected: HSCs (CD34+ HSC), megakaryocyte-erythroid progenitors (CD34+ MEP), early erythroid progenitors (CD34+ ERP-early), erythroid progenitors (CD34+ ERP), immature erythroblasts (early-erythroblast), and mature erythroblasts. During this differentiation pathway, we observed that intercellular entropy increases to reach a maximum at the junction between MEP and early erythroid progenitors and then falls below baseline in the mature erythroblast population (Fig. 1A).

For granulopoiesis, 3440 cells were ordered as follow: HSCs, cycling HSCs (CD34+ HSC-cycle), multi-lineage progenitors (CD34+ MultiLin), granulocytic progenitors (CD34+ Gran), immature neutrophils, and finally, mature neutrophils. The measurement of intercellular entropy revealed a peak occurring at the multi-lineage progenitor step followed by a decrease to a minimum at the latest stages of differentiation (Fig. 1B).

Regarding dendritic differentiation, 699 cells were ordered as follows: HSCs, cycling HSCs, multi-lineage progenitors, lympho-myeloid progenitors (CD34+ LMPP), mono-dendritic progenitors type 2 (CD34+ MDP-2), mono-dendritic progenitors type 1 (CD34+ MDP-1), and finally, mature dendritic cells (dendritic cell). During dendritic cell differentiation, intercellular entropy increased to a maximum at the junction between the cycling HSC populations and multi-lineage progenitors. Then, intercellular entropy decreased to a minimum in mature dendritic cells (Fig. 1C).

For B lymphopoiesis, the analysis focused on the sub-populations which differentiate in the bone marrow, excluding cells maturing in the lymph nodes and home back to the bone marrow (follicular and plasma cells). The 1161 cells were ordered along the pseudotime as follows: HSCs, cycling HSCs, multi-lineage progenitors, lympho-myeloid progenitors, common lymphoid progenitors (CD34+ CLP), cycling pre-B progenitors (CD34+ pre-B cycling), pro-B progenitors (CD34+ pro-B), and pre-B progenitors (CD34+ pre-B). During B lymphocyte maturation, intercellular entropy increased to reach its peak at the level of cycling HSC; a second peak was then observed at the junction between multi-lineage progenitors and lympho-myeloid progenitors (CD34+ LMPP). Intercellular entropy finally decreased to a minimum in the pre-B cells (Fig. 1D).

Importantly, we confirmed the existence of peaks of intercellular entropy on another available scRNA-seq dataset established on 24,088 cells obtained from another healthy bone marrow (HBM2) [25, 28] (Additional file 1: Fig. S3, Table S1). We also confirmed that the presence of the intercellular entropy peak does not depend on the size of the sliding window (Additional file 1: Fig. S4).

Altogether, these analyses indicate that during normal human hematopoiesis, a transient increase of cell-to-cell gene expression variability is a common feature of all the major hematopoietic differentiation pathways.

### Variation in entropy and variation in expression during differentiation highlight different sets of genes

Having observed that stochastic gene expression follows the same dynamic in all the differentiation pathways, we wanted to understand what is driving this intercellular entropy peak. For each gene, we defined “delta-entropy” as the difference between the maximum and the minimum intercellular entropy during a given differentiation pathway. As for the entropy calculation, we calculated the mean expression of each gene on a sliding window of 50 cells moving along the pseudotime, with a step of 10 cells; we defined “delta-expression” as the difference between the maximum and the minimum mean gene expression during differentiation (Additional file 1: Fig. S5).

**Fig. 2:**
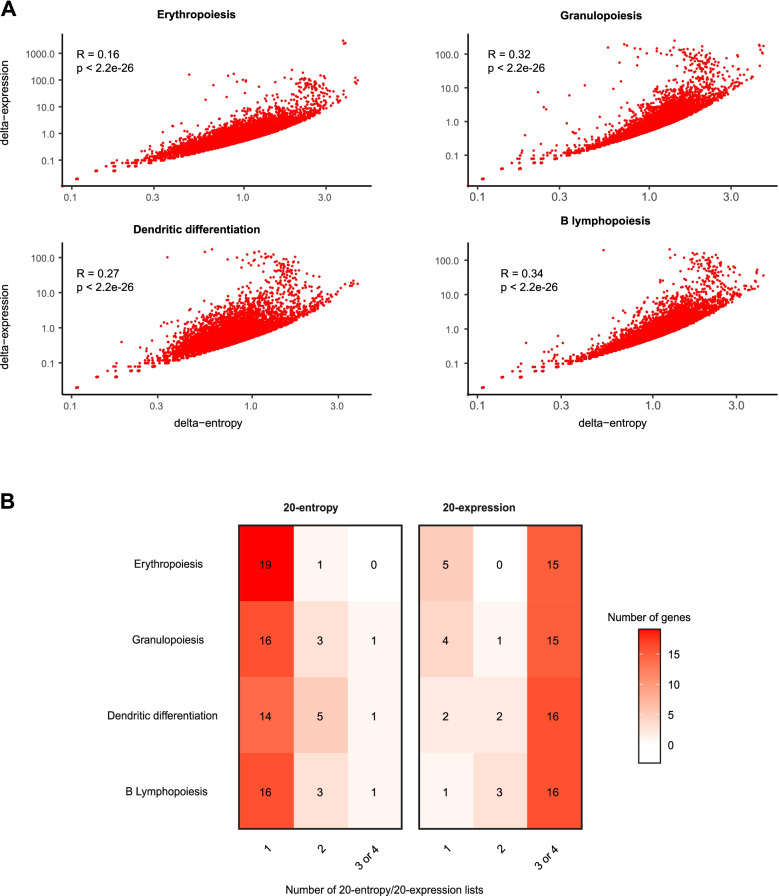
Delta-entropic and delta-expressed genes along hematopoietic differentiation (HBM1). **A** For each gene (red dots on the graphs), delta-expression is represented as a function of delta-entropy (logarithmic scale), in the 4 different hematopoiesis differentiation pathways. **B** Overlay between the different lists. Among the 20 genes that are the most delta-entropic within the erythropoietic pathway, only 1 was also appearing in the most delta-entropic in another differentiation pathway. On the contrary, among the 20 genes with the highest delta-expression in the granulopoiesis pathway, 15 were also appearing in the 20-expression lists in at least two other differentiation pathways

Intuitively, it could be hypothesized that the genes with a high level of delta-expression are the ones with the highest delta-entropy. Indeed, we observed a significant correlation (*p* < 2.10^−16^) with a moderate intensity (*r* < 0.34) between delta-expression and delta-entropy for each differentiation pathway (Fig. 2A). What is worthy of note is that this correlation was markedly reduced for some genes, which prompted us to further analyze the set of genes with the highest delta-entropy or delta-expression. For each differentiation pathway, we selected the 20 genes with the highest delta-entropy (20-entropy) and the 20 genes with the highest delta-expression (20-expression). While most of the genes in the 20-entropy lists were specific to each differentiation pathway, the 20-expression lists were highly similar, with 14 genes common to all lineages (common 20-expression genes) (Fig. 2B, Additional file 1: Fig. S6). Importantly, the same observations were made on the previously studied dataset, highlighting the same specific lists of genes (Additional file 1: Fig. S7, Fig. S8).

Altogether, these analyses show that during normal hematopoiesis, the highest delta-entropic genes are specific to each hematopoietic differentiation pathway, whereas most of the highest delta-expression genes are common to all differentiation pathways.

### Different functions for delta-entropic and delta-expressed genes

**Fig. 3:**
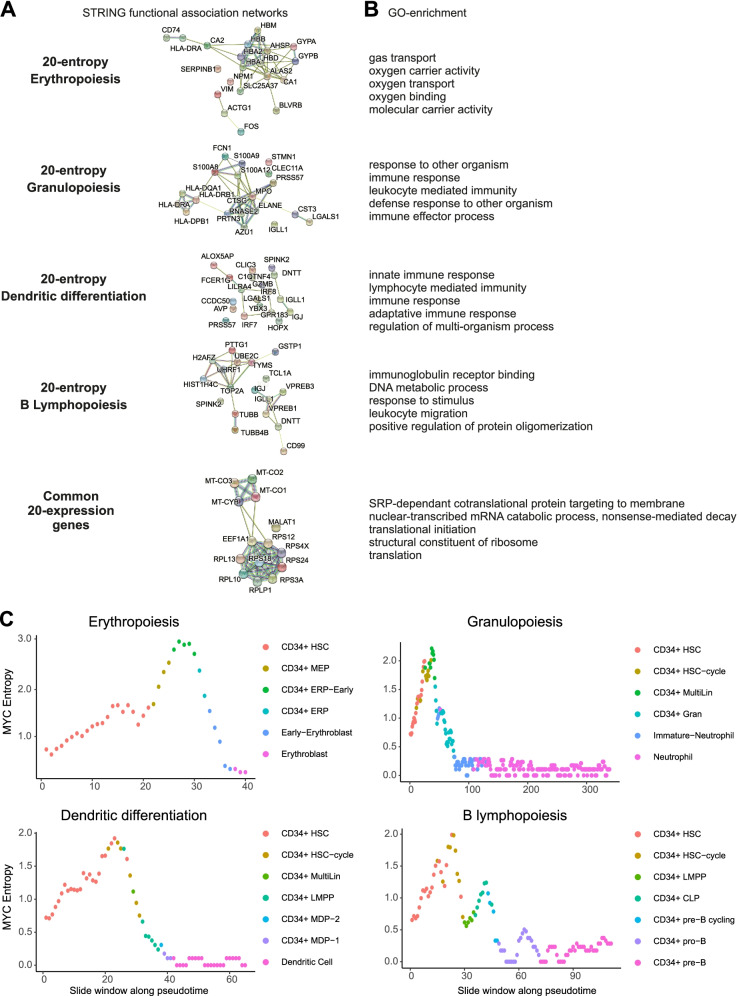
Functional association network and functional enrichment studies of 20-entropy and 20-expression gene lists. Analysis of the interaction networks (**A**) and GO functional enrichment (**B**) of the 20-entropy gene lists and common 20-expression genes with STRING algorithm. For each pathway, only the first five GO terms with a false discovery rate (FDR) lower than 0.05 were represented. **C** Cell-to-cell MYC expression variability during the main pathways of normal hematopoietic differentiation (HBM1)

We used the STRING database [29] to analyze the interaction network and functional GO enrichment of the 20-entropy gene lists and common 20-expression genes. For each gene list, we showed strong functional interactions between genes (Fig. 3A). Moreover, the functional GO enrichment (biological process and molecular function) in each gene list highlighted specific functions and processes of the corresponding differentiation pathway (e.g., oxygen transport for erythropoiesis) (Fig. 3B).

More precisely, the 20-entropy list for erythropoiesis included 9 genes involved in hemoglobin synthesis (*HBA1*, *HBA2*, *HBB*, *HBD*, *HBM*, *BLVRB*, *ALAS2*, *SLC25A37*, *AHSP*) and 4 genes involved in other important erythropoietic processes (*CA1*, *CA2*, *GYPA*, *GYPB*). Similarly, the 20-entropy list for granulopoiesis included alarmin genes (*S100A8*, *S100A9*, *S100A12*), genes encoding for antibacterial and antiviral proteins (*AZU1*, *MPO*, *PRTN3*, *ELANE*, *CTSG*, *CST3*, *RNASE2*), antigen-presenting molecules (*HLA-DPB1*, *HLA-DQA1*, *HLA-DRB1*), and lectins (*FCN1*, *CLEC11A*). For dendritic maturation, the list contained genes regulating the response to interferon and markers of mature dendritic populations and progenitors (*IRF7*, *IRF8*) and genes playing a role in the inflammatory response (*ALOX5AP*) and in innate immunity (*GZMB*, *C1QTNF4*). For B lymphopoiesis, the 20-entropy list comprised critical genes of B cell development such as pre-BCR formation (*VPREB1*, *VPREB3*), and immunoglobulin light chains (*IGLC2*, *IGLC3*). Interestingly, the common 20-expression genes encoded for the protein translation machinery (*RPS4X*, *RPL13*, *RPLP1*, *RPL10*, *RPS24*, *RPS12*, *RPS18*, *RPS3A*, *EEF1A*) and the mitochondrial respiratory chain (*MT-CO1*, *MT-CO2*, *MT-CO3*, *MT-CYB*). The long non-coding RNA (MALAT1) [30] was also on the common 20-expression list.

Again, very similar results were obtained on the second dataset retrieved from the healthy bone marrow (HBM2).

Altogether, these analyses show that the highest delta-entropic genes are not only specific to each hematopoietic differentiation pathway but are also known to play a major role in specific functions and processes of these pathways. On the contrary, most of the highest delta-expressed genes are common to all differentiation pathways, encoding proteins essential for cell survival and proliferation.

Among all the genes, those encoding transcription factors are especially important given their ability to influence the expression of a large number of genes. We conducted an analysis of the variation of the intercellular entropy of the genes identified as bona fide transcription factors. For each differentiation pathway, we selected transcription factors that belonged to the top 1000 delta-entropic genes (Additional file 1: Fig. S9, Fig. S10, Fig. S11, Fig. S12). We showed that transcription factors tend to have a peak of cell-to-cell gene expression variability along all four differentiation pathways. Moreover, *MYC* intercellular entropy seemed to be consistent with the mean intercellular entropy of all genes in the four differentiation pathways (Fig. 3C).

### A peak in cell-to-cell gene expression variability is also observed during hematopoiesis in healthy elderly subjects and SF3B1-mutated MDS

To further assess the role of cell-to-cell gene expression variability in pathological hematopoiesis differentiation, we reasoned that myelodysplastic syndromes (MDS) would be an interesting model. Indeed, low-risk MDS are characterized by impaired differentiation and excessive cell death of progenitors, leading to peripheral cytopenia [31, 32]. Accordingly, we hypothesized that the analysis of the variations of cell-to-cell gene expression during differentiation could identify the differences between MDS and controls. Especially for this study, we obtained and used CD34+ hematopoietic stem and progenitor cells (HSPCs) from two healthy elderly controls (Ctrl1, Ctrl3) and two *SF3B1* mutated MDS patients at diagnosis (MDS2, MDS4) (Additional file 1: Table S2). We generated transcriptomic data of 12,689 HSPC distributed over our 4 samples [33] (Additional file 1: Table S3). In order to avoid bias due to the differences in cell number in the late stages of differentiation, we focused our experiments on the CD34+ HSPC compartment, at the root of all differentiation sequences (Additional file 1: Fig. S13A).

We generated a reference map of all cells from the 4 samples with the integration method implemented in Seurat [25]. The resulting UMAP allowed us to distinguish 21 different cell subtypes that are organized according to the major hematopoietic differentiation pathways (erythropoiesis, granulopoiesis, dendritic differentiation, and B lymphopoiesis) (Additional file 1: Fig. S13B, Fig. S13C). The specific markers of the sub-populations identified by SingleR were consistent with previous studies of the bone marrow HSPC compartment [24, 26, 34] (Additional file 1: Fig. S14).

In order to compare the intercellular entropy variations between MDS patients and controls, we used the integrated gene-cell matrix of the 4 samples to calculate a common pseudotime. For each differentiation pathway, we performed a subsampling to analyze the same cell number, in each cell type, for each patient. Intercellular entropy was then computed for each patient over a sliding window of 50 cells advancing with a step of 10 on the common pseudotime**.**

**Fig. 4:**
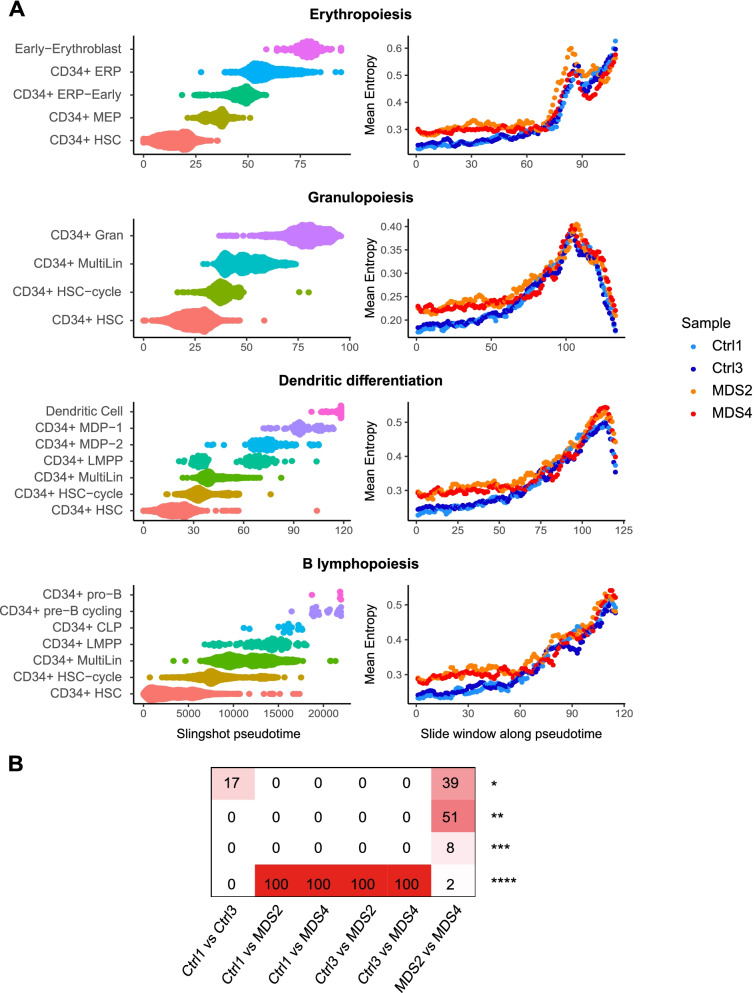
Evolution of cell-to-cell gene expression variability during hematopoiesis in elderly subjects and SF3B1-mutated MDS. **A** For each differentiation pathway, a common pseudotime was calculated on the integrated gene cell matrix of the 4 samples. A sub-sampling was performed to have the same number of cells in each cell type per sample. The average intercellular entropy of all genes was then calculated individually for each patient on a sliding window of 50 cells advancing with a step of 10 cells on the common pseudotime. **B** Intercellular entropy of all genes was calculated on a subsample of 700 HSCs of healthy elderly patients and SF3B1-mutated MDS. A Wilcoxon assay was used to compare the mean intercellular entropy between samples. This was repeated 100 times. Shown is the number of times the resulting test gave a certain level of *p*-value: *p < 0.05, **p < 0.01, ***p < 0.001, ****p < 0.0001. In 100% of the subsamples, the difference in the mean intercellular entropy between control and MDS patients was very highly significant (*p* < 0.0001)

After applying this method, a peak of intercellular entropy was observed in healthy subjects and MDS for all routes of differentiation (Fig. 4A)

For granulopoiesis, dendritic differentiation, and B lymphopoiesis, intercellular entropy peaked in the population of multi-lineage progenitors and then declined in the more mature populations (Additional file 1: Fig. S15, Fig. S16, Fig. S17). For erythropoiesis, no decrease was observed, probably because of the lack of sufficient numbers of mature erythroid cells due to preanalytical steps (CD34 cell sorting) (Additional file 1: Fig. S18).

We then compared the top 100 delta entropic genes, in each hematopoietic differentiation pathway, for each MDS and control sample (Additional file 1: Fig. S19). We showed that in each pathway, the majority of delta entropic genes are common between the four samples (Additional file 1: Table S4) and that some delta entropic genes are specific to MDS (Additional file 1: Fig. S19).

When observing the pattern of cell-to-cell gene expression variability in healthy elderly and MDS patients, we noted that intercellular entropy of the cells at the root of all differentiation pathways was higher in MDS than in healthy elderly subjects (Fig. 4A). To confirm this observation, we performed 100 random subsampling of 700 HSCs in each sample and then computed the mean intercellular entropy of all genes. No difference was found between the mean intercellular entropies of HSCs of the two healthy elderly subjects. However, the HSC intercellular entropy of MDS patients was significantly higher than the one of healthy elderly subjects (Fig. 4B).

These data suggest that cell-to-cell gene expression variability is increased in MDS HSCs when compared to healthy age-matched controls.

## Discussion

Differentiation can be defined as the progressive acquisition of phenotypic differences enabling the production of highly specialized mature cells from a pool of stem cells. The driving forces of differentiation have been described according to two theoretical frameworks, highlighting the role of cell-extrinsic stimuli either to initiate the process (instructive model) or to select random priming of differentiation (stochastic model) (see, e.g., [5, 12, 14, 35–37] for elements of the discussion). Interestingly, the prediction of the evolution of cell-to-cell variability during differentiation is very different, depending on the model. According to the instructive model, cell-to-cell phenotypic variability should be limited and should not vary during differentiation, since all the cells committed to a given lineage are following the same instructions. On the contrary, the stochastic model predicts that cell-to-cell variability should increase to reach a maximum at the stage where the selection occurs. With the technological outbreak of single-cell RNA-seq, we can now use cell-to-cell gene expression variability as a proxy of cell-to-cell phenotypic variability and provide new insights into the historical controversies between the two models of differentiation.

Using hematopoiesis as a model, we computed the Shannon entropy to measure cell-to-cell gene expression variation. It is noteworthy that the use of Shannon entropy to analyze the scRNA-seq data can be equivocal. Some authors measure the variability of gene expression in a single cell (what we called intracellular entropy) which has been shown to be a proxy for stemness [38, 39]. With this approach, the differentiation process is associated with a continuous decrease in entropy, because the transcriptional program is getting more and more specialized as cells undergo differentiation. In the present study, our aim was to capture cell-to-cell variability by measuring intercellular entropy to quantify the transcriptomic differences between cells at the same stage of differentiation.

The first important conclusion of this study is that a peak of entropy, based on cell-to-cell gene expression variation, is observed during the differentiation of hematopoietic stem cells along each lineage studied, strongly supporting the stochastic view of differentiation. This is in full agreement with Moussy et al. [40] which demonstrates that human HSCs grown in vitro do exhibit a phase characterized by fluctuating phenotypic behavior. However, the data presented here do not demonstrate any causal role of cell-to-cell gene expression variability during differentiation. This essential question could be addressed using pharmacological agents with the capacity to modify the level of stochastic gene expression [15, 18].

Alternatively, a recent paper has reported the results of a screen which identify genes modifying cell-to-cell variability in a melanoma cell line [41]: assessing how the knockout of these genes impacts the shape of the intercellular entropy in hematopoietic differentiation and the outcome of hematopoiesis in animal models could be very interesting.

Notably, many biological mechanisms can buffer stochastic gene expression and reduce the phenotypic impact of the differences observed at the mRNA level [42–45]. Indeed, the half-life of proteins being significantly longer could buffer the variability observed at the mRNA level [46]. Solid single-cell proteomic data would represent an important step to assess the extent to which measurable features at the mRNA level might be a good proxy of phenotypic variations.

Although we observed a statistically significant correlation between variation in the gene expression level and variation in intercellular entropy occurring during the course of a given differentiation pathway, the magnitude of this correlation remained low.

The genes with the strongest intercellular entropy variation along a given differentiation pathway were enriched in genes known to be associated with this particular lineage. By contrast, the genes with the strongest variation in the mean expression level were shared by all differentiation pathways and contained genes encoding proteins involved in ribosome biogenesis, protein translation, or mitochondrial respiration, essential mechanisms of cell survival. These genes are commonly described as essential in whole-genome knock-out screens [47, 48], so we can hypothesize that stochastic variations in their expression are not compatible with cell survival, explaining why we do not measure high entropy variation for these genes. Moreover, their expression could be less noisy because of redundancy or other noise buffering systems [1]. By contrast, the most highly entropic genes were very specific to each differentiation pathway. Strikingly, these specific lists precisely comprised genes already described in the differentiation pathway in question, including specific transcription factors, which enforces the biological signification of this observation.

Given the preeminent impact of differentiation abnormalities in myelodysplastic syndromes (MDS), we also assessed cell-to-cell gene expression variability in the bone marrow of two low-risk MDS patients. In this pathological hematopoiesis, the shape of the intercellular entropy variation during differentiation was conserved. Concerning the most delta entropic genes, even if some genes are specific to MDS, we believe that a 2 vs 2 comparison is not sufficient to conclude anything about their role in MDS pathophysiology. Interestingly, we observed a significant increase in entropy in the stem cell compartment in comparison with age matched-control samples. These data suggest that enhanced cell-to-cell gene expression variability in the HSC compartment in MDS could be an interesting feature of this pre-leukemic process.

We have considered the possibility that the computational annotation of HSCs from healthy donors versus MDS patients could be affected by differences in the “healthy vs diseased” transcriptional states of the two groups of individuals. Because our annotation with SingleR is based on healthy donor references, this could introduce a substantial bias in the analysis. However, it has been shown in CD34+ HSPCs that gene expression information alone is sufficient to discriminate between stem cells and progenitors, but insufficient to distinguish cancerous from non-cancerous cells and that the mutational status is essential to accurately identify this difference [49]. Accordingly, we do not think that the differences observed in cell-to-cell variability can be totally explained by artifact(s) due to the application of SingleR to annotate cells from MDS samples.

The two MDS patients analyzed were both carriers of the *SF3B1* mutation. Importantly, we and others have described *SF3B1* mutations as heterozygous and showed that the variant allele frequency (VAF) detected in bone marrow mononuclear cells represent the clonal architecture of the HSPC compartment [50]. Therefore, we could only infer that in these samples, the *SF3B1*-mutated population represents 70 and 86% of the total population respectively. We hypothesized that even if the majority of HSCs are *SF3B1* mutated, coexistence with non-mutated HSCs could be at least partly responsible for the increased cell-to-cell variability compared to healthy subjects.

Since *SF3B1* mutations affect the splicing machinery, it could be interesting to calculate for each gene the Shannon entropy of transcript isoforms in order to assess the level of “splicing entropy.” In our work, we have used 10× genomics 3′ short-read sequencing, which does not allow optimal characterization of the alternative transcripts, but this may be possible with a technology that couples single-cell genotyping of *SF3B1*-mutated MDS with long-read transcriptome sequencing [51].

This technology could also allow us to separately assess entropy variation in both *SF3B1*-mutated and WT hematopoiesis in the same bone marrow sample, and could be useful, in the future, to explore the role of specific mutations in cell-to-cell variability.

Further studies are warranted to explore to what extent the increase of cell-to-cell variability in the stem cell compartment is contributing to MDS pathophysiology, as well as the specific role of the *SF3B1* mutation in this process.

## Conclusions

The first conclusion of this study is that a peak in cell-to-cell gene expression variation is observed during the differentiation of hematopoietic stem cells along each lineage studied, strongly supporting the stochastic view of differentiation. Thus, our study completely supports the wealth of recent studies highlighting the intensity and importance of gene expression stochasticity in all systems examined to date [52–56]. Finally, we showed that the level of cell-to-cell variation is increased in the most immature compartment of hematopoiesis in myelodysplastic syndromes. More generally, this study highlights the need to consider the role of stochastic gene expression in complex physiological processes and pathologies such as cancers [57, 58] paving the way for possible noise-based therapies through epigenetic regulation [59].

## Methods

### Human samples

Patients Ctrl1 (female, 68 years old) and Ctrl3 (male, 74 years old) were healthy elderly subjects with normal blood counts and not taking any medication that may affect hematopoiesis (chemotherapy or immunosuppressants). All patients provided written informed consent. Control samples were obtained after the explanation of the study by the surgeon and the signature of a non-opposition consent. MDS2 and MDS4 patients displayed MDS with ring sideroblasts mutated for the SF3B1 gene. Both patients signed the OncoCentre Consent.

The bone marrow samples from healthy elderly patients (Ctrl1 and Ctrl3) were obtained by extraction of the bone marrow cells from the bone of the femoral head. Femoral heads were obtained after informed consent, during a surgery for hip replacement. They were cut in half and collected in a conservation medium (Hanks balanced salt solution with NaHCO3, Eurobio™) supplemented with heparin and then transported to the laboratory at room temperature. The femoral heads were then scraped with a spatula, grounded in a mortar, and washed with a PBS solution supplemented with DNAse at 100 μg/ml. The mononuclear cells were finally isolated on a Ficoll gradient.

The bone marrow samples from patients with myelodysplastic syndromes (MDS2, MDS4) were obtained following a bone marrow aspiration performed as part of the disease diagnosis determination. The mononuclear cells were isolated from the samples on a Ficoll gradient, counted, and resuspended in an adequate solution (IMDM, SVF 40%, DMSO 15%) before being frozen in liquid nitrogen at a concentration of 20 to 30 million cells per mL. The clinical and biological characteristics of the patients are summarized in Additional file 1: Table S2.

### Genomics studies

Bone marrow mononuclear cells were purified on Ficoll gradients, and pellets were processed for nucleic acid extraction using a DNA/RNA Kit (Qiagen). Genomic DNA was studied by high-throughput sequencing of 45 genes recurrently mutated in myeloid malignancies using a panel designed on Human genome hg19, and sequencing was performed on Ion PGM™ (Life Technologies) on a dedicated 318 V2 chip [60]. Libraries were prepared using Ion AmpliSeq library kit2 384 (Life Technologies) according to the manufacturer’s instructions. The average coverage per gene was ≥ 500×. Reads were aligned against human genome build 19 (hg19) and analyzed for single nucleotide variant (SNV) calling with the NextGENe software (SoftGenetics, Chicago, IL) and with an in-house pipeline (Polydiag, Institut Imagine, Université de Paris). We reported all clinically relevant variants with a variant allele frequency (VAF) cutoff at 2%. All the samples were also screened for ASXL1 (including c.1934dupG; p.G646WfsX12) and SRSF2 mutations by Sanger sequencing. Moreover, aligned reads from .bam files were visualized using the Integrative Genomics Viewer v2.3 from the Broad Institute (Cambridge, MA, USA). Assessment of variant implication was performed based on population databases (dbSNP and GnomAD), mutation databases (COSMIC), and prediction software (Alamut, mutation taster, OncoKB, and Cancer Genome Interpreter).

### Single-cell RNA-seq

Bone marrow mononuclear cells were thawed, and dead cells were eliminated by immunomagnetic negative sorting (MACS MicroBead technology from Miltenyi Biotec™). A second CD34-positive immunomagnetic sorting was performed to isolate stem and progenitor cells. Cells were then washed with PBS containing 0.04% BSA. Cell concentration and viability were determined microscopically with a Malassez counting chamber cell after staining with trypan blue.

Libraries were prepared with the chromium system of 10× genomics, with the Chromium Single Cell 3’ V2 kit according to the manufacturer’s protocol (www.10xgenomics.com). The four samples (Ctrl1, Ctrl3, MDS2, and MDS4) were processed on the same chromium chip. The number of cells targeted per sample was 5000. The libraries were sequenced by the Integragen company on an Illumina HiSeq4000 sequencer with a target depth of 50,000 reads per cell. Sequencing data are deposited in the Gene Expression Omnibus (GEO) with the accession code GSE169426 [33].

### Bioinformatic analysis

#### Gene-cell expression matrices

Healthy donor total bone marrow scRNA-seq datasets used in this study were published by Granja et al. (HBM1) [19, 20] and Stuart et al. (HBM2) [25, 28].

For HBM1, gene-cell expression matrix files were downloaded from https://www.ncbi.nlm.nih.gov/geo/query/acc.cgi?acc=GSE139369 (GSM4138872_scRNA_BMMC_D1T1.rds.gz, GSM4138873_scRNA_BMMC_D1T2.rds.gz). Since the authors removed the ribosomal and mitochondrial genes from the gene-cell matrix, the original .bam files were downloaded from https://trace.ncbi.nlm.nih.gov/Traces/sra/?run=SRR10343065 (SRR10343065/scRNA_BMMC_D1T1.bam.1) and https://trace.ncbi.nlm.nih.gov/Traces/sra/?run=SRR10343066 (SRR10343066/scRNA_BMMC_D1T2.bam.1) and therefore processed using the DropEst [61] software. The expression of the mitochondrial and ribosomal genes obtained was then reincorporated into the previously filtered gene-cell matrix.

For HBM2, gene-cell expression matrix files were downloaded from https://www.ncbi.nlm.nih.gov/geo/query/acc.cgi?acc=GSM3681518 (GSM3681518_MNC_RNA_counts.tsv.gz) and https://www.ncbi.nlm.nih.gov/geo/query/acc.cgi?acc=GSM3681520 (GSM3681520_MNC_HTO_counts.tsv.gz).

For Ctrl1, Ctrl3, MDS2, and MDS4 samples, the Cell Ranger software (https://support.10xgenomics.com/single-cellgene-expression/software/pipelines/latest/what-is-cell-ranger) was used to process the raw data from Illumina sequencing and to generate the gene-cell expression matrices. The reads were aligned on the GRCh38 reference genome by STAR with the ENSEMBL annotation.

#### Gene expression matrix filtering

For HBM1, we kept the same filters as the authors did [19].

For HBM2 downstream analysis, we chose to keep the cells expressing between 500 and 4000 genes with a percentage of mitochondrial genes lower than 15%.

For Ctrl1, Ctrl2, MDS2, and MDS4, we chose to keep, for downstream analysis, the cells which expressed between 500 and 5500 genes with a percentage of mitochondrial genes lower than 10%.

#### Dimensionality reduction

The gene-cell expression matrices of each sample were normalized with SCtransform [62]. After normalization, the gene-cell matrices were subjected to dimensionality reduction techniques such as principal component analysis (PCA) and UMAP [63] using Seurat [25]. We also used the Scanpy [64] python package to calculate the ForceAtlas2 (FA). For HBM1 and HBM2, we chose not to use ribosomal and mitochondrial genes in order to improve the results.

#### Cell annotation

To perform the cell annotation, we did not use any unsupervised clustering algorithm. The cells were annotated individually using the SingleR [22] software by comparing their gene expression profile with the 34 bone marrow populations published by Hay et al. [23].

#### Pseudotime ordering

For each individual sample, the cells were classified from HSCs to mature cells for each differentiation pathway. A pseudotime was computed with Slingshot [27] with the following options: the starting cell population was always specified as CD34 + HSC; the multidimensional space specified was either the UMAP, the FA, or the PCA; and the final pseudotime cell population was sometimes specified as the sub-population corresponding to the most mature cells of the differentiation pathway studied. Thus, depending on the options chosen, several pseudotimes were calculated for each differentiation path. The pseudotime chosen for intercellular entropy analyses was the one allowing the cells to be ordered for each differentiation pathway in the most consistent way regarding our knowledge of hematopoiesis.

For Ctrl1, Ctrl3, MDS2, and MDS4, gene-cell expression matrices were integrated using Seurat [25] after normalization with SCtransform technique in order to compute common multidimensional spaces (PCA, UMAP, and FA). This allowed Slingshot to compute a common pseudotime on the four differentiation pathways.

#### Intercellular entropy computation

For each sample, the intercellular entropy was computed on the raw counts of the filtered gene expression matrices for each differentiation pathway. We computed an intercellular Shannon entropy per gene and displayed the mean entropy variation along the differentiation sequence. Since we average this value, it will not be affected by the number of genes which may vary as a function of time.

Because Ctrl1 and Ctrl3 cells come from samples that were mechanically dissociated, we chose not to include “dissociation genes” for the comparison of intercellular entropy between elderly subjects and *SF3B1*-mutated MDS. These “dissociation genes” were the common genes between the two dissociation signatures previously published [65, 66].

#### Shannon entropy estimation

To estimate the Shannon entropy, the best upper bound estimator (BUB) was computed as developed in Paninski et al. [67]. The *N*/*m* ratio is critical for the choice of the proper entropy estimator, with *N* being the number of cells used to compute the entropy, and *m* being the number of bins, here the largest number of RNA molecules found in each cell. For small *N*/*m* ratios (less than 100), the BUB was shown to outperform the maximum likelihood estimator, the Miller-Madow, or the Jackknifed estimator, by minimizing the maximum error. Since we are using sliding windows of 50 cells, the choice of the BUB estimator prevailed. The R script for this estimation is available on this OSF project: https://osf.io/9mcwg/

## Supplementary Information

**Additional file 1: Fig. S1.** Single cell transcriptomic landscape of healthy human bone marrow (HBM1). **Fig. S2.** Strategy used to represent the evolution of cell-to-cell gene expression variability during differentiation. **Fig. S3.** Evolution of cell-to-cell gene expression variability during the main pathways of normal hematopoietic differentiation (HBM2). **Fig. S4.** Test of different size for the sliding window*.* **Fig. S5.** Strategy used to calculate delta-entropy and delta-expression. **Fig. S6.** Most delta-entropic and most delta-expressed genes along hematopoietic differentiation (HBM1). **Fig. S7.** delta-entropic and delta-expressed genes along hematopoietic differentiation (HBM2). **Fig. S8.** Most delta-entropic and most delta-expressed genes along hematopoietic differentiation (HBM2). **Fig. S9.** Cell-to-cell gene expression variability of transcription factors belonging to the 1000 most delta entropic genes during Erythropoiesis (HBM1). **Fig. S10.** Cell-to-cell gene expression variability of transcription factors belonging to the 1000 most delta entropic genes during Granulopoiesis (HBM1). **Fig. S11.** Cell-to-cell gene expression variability of transcription factors belonging to the 1000 most delta entropic genes during dendritic differentiation (HBM1). **Fig. S12.** Cell-to-cell gene expression variability of transcription factors belonging to the 1000 most delta entropic genes during B lymphopoiesis (HBM1). **Fig. S13.** Transcriptional landscape of the HSPC compartment of SF3B1 mutated MDS and healthy elderly subjects. **Fig. S14.** Expression values of selected marker genes for all cell sub-populations of the HSPC compartment of SF3B1 mutated MDS and healthy elderly subjects. **Fig. S15.** Evolution of cell-to-cell gene expression variability during granulopoiesis in elderly subjects and SF3B1-mutated MDS. **Fig. S16.** Evolution of cell-to-cell gene expression variability during dendritic differentiation in elderly subjects and SF3B1-mutated MDS. **Fig. S17.** Evolution of cell-to-cell gene expression variability during B lymphopoiesis in elderly subjects and SF3B1-mutated MDS. **Fig. S18.** Evolution of cell-to-cell gene expression variability during Erythropoiesis in elderly subjects and SF3B1-mutated MDS. **Fig. S19.** Comparison of the 100 most delta entropic genes along hematopoietic differentiation between MDS patients and age-matched healthy subjects. **Table S1.** Distribution of cellular subpopulations for each differentiation pathway in HBM1 and HBM2 dataset. **Table S2.** Clinico-biological features of patients from whom HSPCs were harvested for the scRNA-seq experiment. **Table S3.** Distribution of cellular subpopulations for each differentiation pathway in Ctrl1, Ctrl3, MDS2 and MDS4 samples. **Table S4.** Common delta-entropic genes between MDS patients and age-matched healthy subjects during the different hematopoietic differentiation pathways.

## Data Availability

Single-cell RNA-seq data are deposited in the Gene Expression Omnibus (GEO) with the accession code GSE169426 [33].

## Abbreviations

BUB: Best upper bound estimator
CLP: Common lymphoid progenitor
ERP: Erythroid progenitor
FA: ForceAtlas2
GEO: Gene Expression Omnibus
Gran: Granulocytic progenitor
HSC: Hematopoietic stem cell
HSPC: Hematopoietic stem and progenitor cell
LMPP: Lympho-myeloid progenitors
MDP: Mono-dendritic progenitor
MDS: Myelodysplastic syndrome
MEP: Megacaryocyte-erythroid progenitor
MultiLin: Multilineage progenitor
PCA: Principal component analysis
scRNA-seq: Single-cell RNA sequencing
UMAP: Uniform Manifold Approximation and Projection for Dimension Reduction
