# Supplementary material for: Hematopoietic differentiation is characterized by a transient peak of entropy at a single-cell level
Source: BMC Biol. 2022 Mar 9;20:60. doi: 10.1186/s12915-022-01264-9 (PMC8905725; doi:10.1186/s12915-022-01264-9)
Supplement: Supplementary file 1 — Additional file 1: Fig. S1. Single cell transcriptomic landscape of healthy human bone marrow (HBM1). Fig. S2. Strategy used to represent the evolution of cell-to-cell gene expression variability during differentiation. Fig. S3. Evolution of cell-to-cell gene expression variability during the main pathways of normal hematopoietic differentiation (HBM2). Fig. S4. Test of different size for the sliding window. Fig. S5. Strategy used to calculate delta-entropy and delta-expression. Fig. S6. Most delta-entropic and most delta-expressed genes along hematopoietic differentiation (HBM1). Fig. S7. delta-entropic and delta-expressed genes along hematopoietic differentiation (HBM2). Fig. S8. Most delta-entropic and most delta-expressed genes along hematopoietic differentiation (HBM2). Fig. S9. Cell-to-cell gene expression variability of transcription factors belonging to the 1000 most delta entropic genes during Erythropoiesis (HBM1). Fig. S10. Cell-to-cell gene expression variability of transcription factors belonging to the 1000 most delta entropic genes during Granulopoiesis (HBM1). Fig. S11. Cell-to-cell gene expression variability of transcription factors belonging to the 1000 most delta entropic genes during dendritic differentiation (HBM1). Fig. S12. Cell-to-cell gene expression variability of transcription factors belonging to the 1000 most delta entropic genes during B lymphopoiesis (HBM1). Fig. S13. Transcriptional landscape of the HSPC compartment of SF3B1 mutated MDS and healthy elderly subjects. Fig. S14. Expression values of selected marker genes for all cell sub-populations of the HSPC compartment of SF3B1 mutated MDS and healthy elderly subjects. Fig. S15. Evolution of cell-to-cell gene expression variability during granulopoiesis in elderly subjects and SF3B1-mutated MDS. Fig. S16. Evolution of cell-to-cell gene expression variability during dendritic differentiation in elderly subjects and SF3B1-mutated MDS. Fig. S17. Evolution of cell-to-cell gene expressio [file 12915_2022_1264_MOESM1_ESM.pdf]

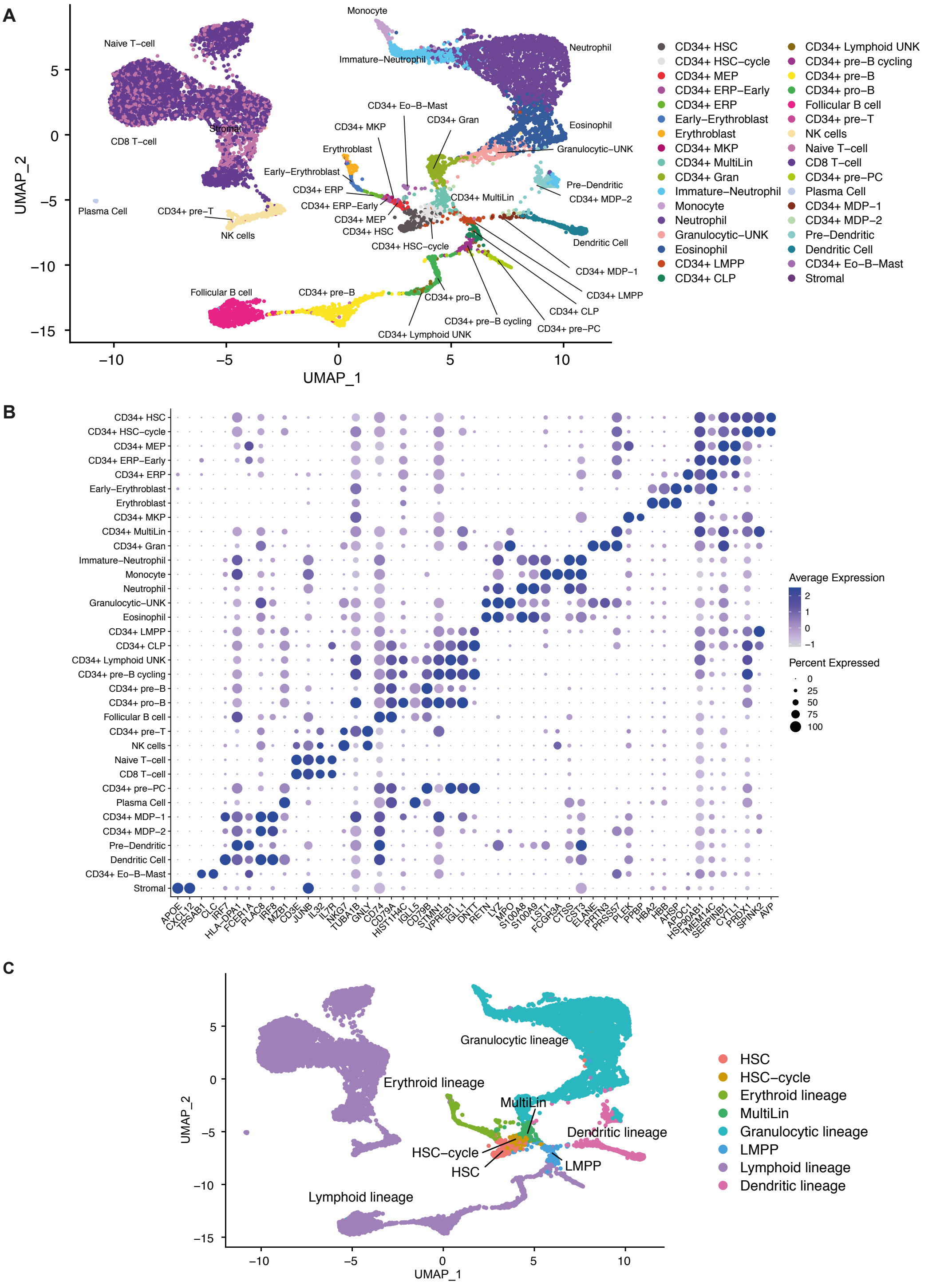

Figure S1: Single cell transcriptomic landscape of healthy human bone marrow (HBM1). A) UMAP of scRNAseq data on healthy donor bone marrow mononuclear cells published by Granja et al (19), 34 of the 35 populations described by Hay et al (22) were distinguished by SingleR. **B)** Expression values of selected marker genes for all cell sub-populations. Circle color shows mean scaled expression values and circle size represents the proportion of expressing cells per sub-populations. **C)** UMAP of Main hematopoietic differentiation pathways (erythropoiesis, granlulopoiesis, B lymphopoiesis and dendritic maturation).

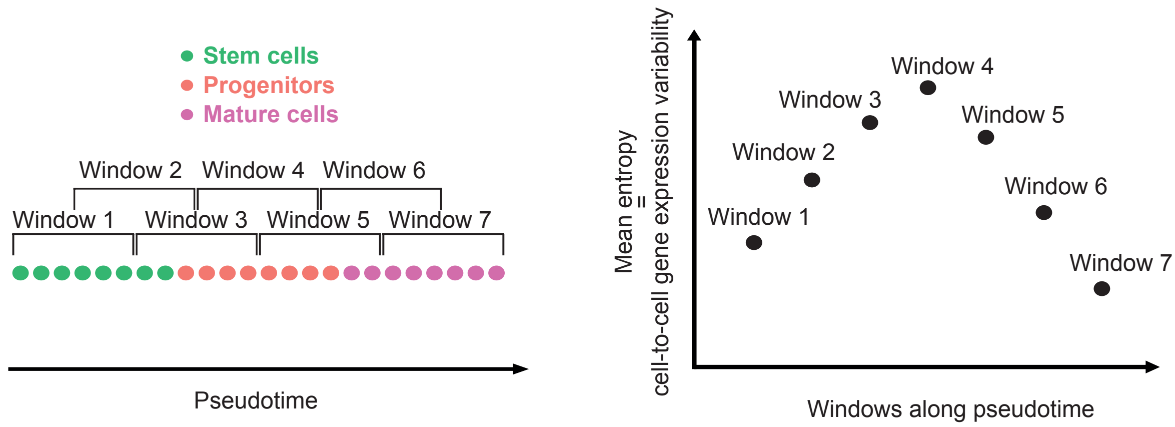

Figure S2: Strategy used to represent the evolution of cell-to-cell gene expression variability during differentiation.

Cells are first ordered according to their position in the pseudotime which reflects their progress across the differentiation. The mean intercellular entropy of all genes was then calculated on a window of (for the example) 6 cells (window1). The window is moving across pseudotime (window2, window3…) with a step of (for the example) 3 cells, and the mean intercellular entropy is calculated for every windows (right panel).

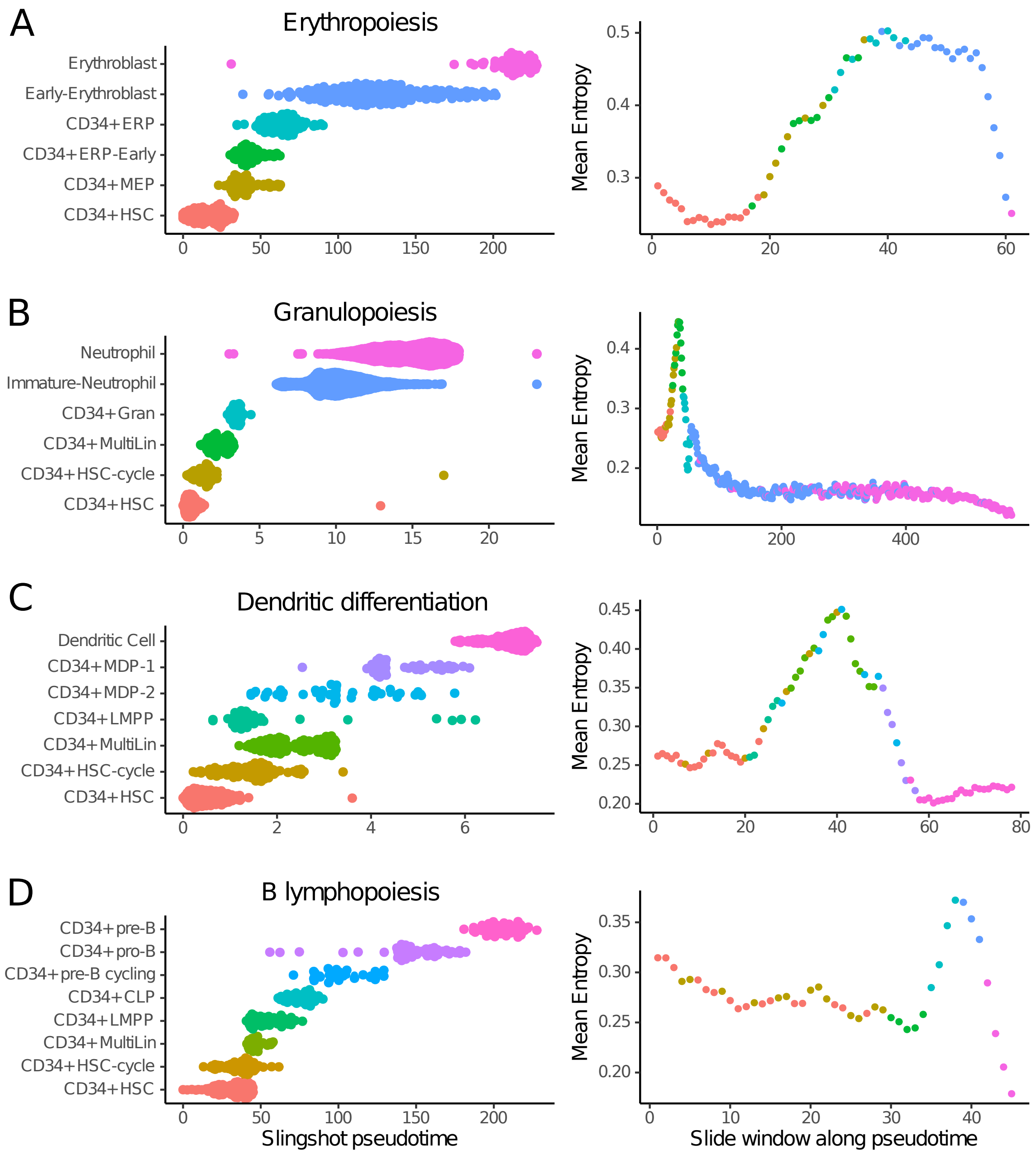

Figure S3: Evolution of cell-to-cell gene expression variability during the main pathways of normal hematopoietic differentiation (HBM2).

Cell populations belonging to each differentiation pathway were first selected and then ordered according to the pseudotime calculated by Slingshot. The average intercellular entropy of all genes was then calculated on a sliding window of 50 cells which moves across the pseudotime with a step of 10 cells (the color of each point on the graph correspond to the nature of the first cell in the corresponding sliding window). **A)** Erythropoiesis **B)** Granulopoiesis **C)** Dendritic differentiation **D)** B Lymphopoiesis

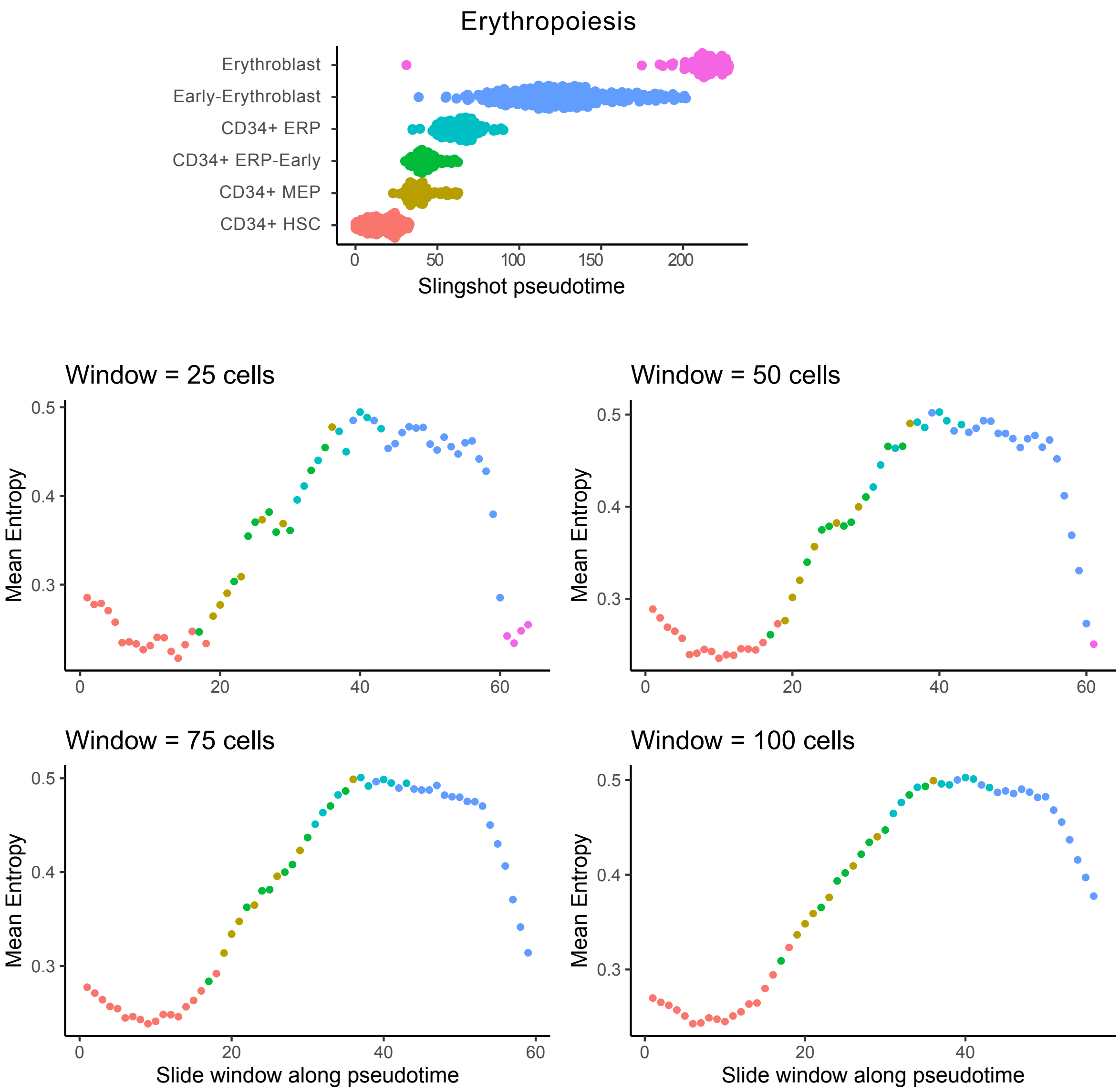

Figure S4: Test of different size for the sliding window.

Example of the evolution of cell-to-cell gene expression variability during normal erythropoiesis (HBM2) with different sliding window sizes.

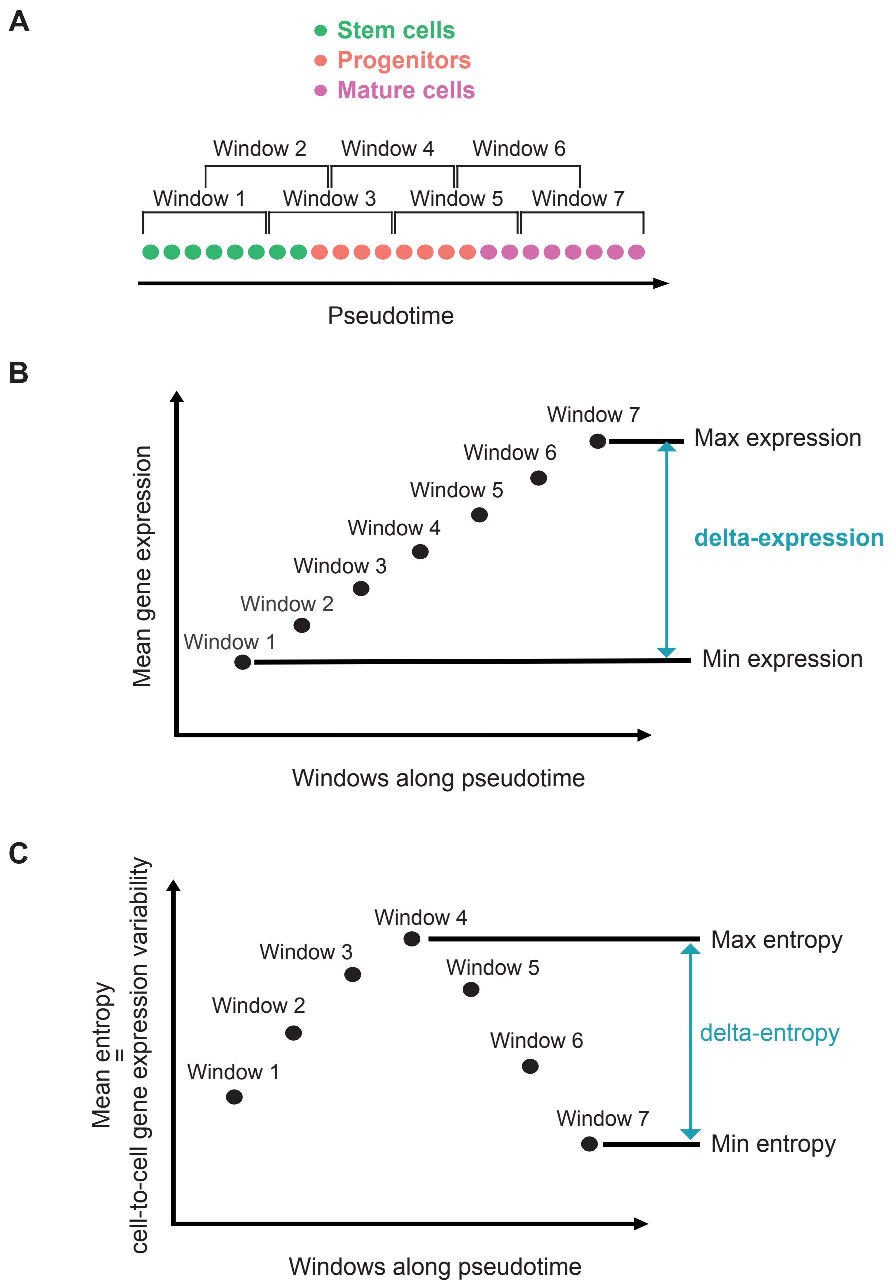

Figure S5: Strategy used to calculate delta-entropy and delta-expression.

(**A**) Cells are first ordered according to their position in the pseudotime which reflects their progress across the differentiation.

(B) The mean expression of a gene is then calculated for each window. For each gene, the difference between minimum and maximum expression is the delta-expression

(C) The intercellular entropy of a gene is also calculated for each window. For each gene, the difference between minimum and maximum entropy is the delta-entropy.

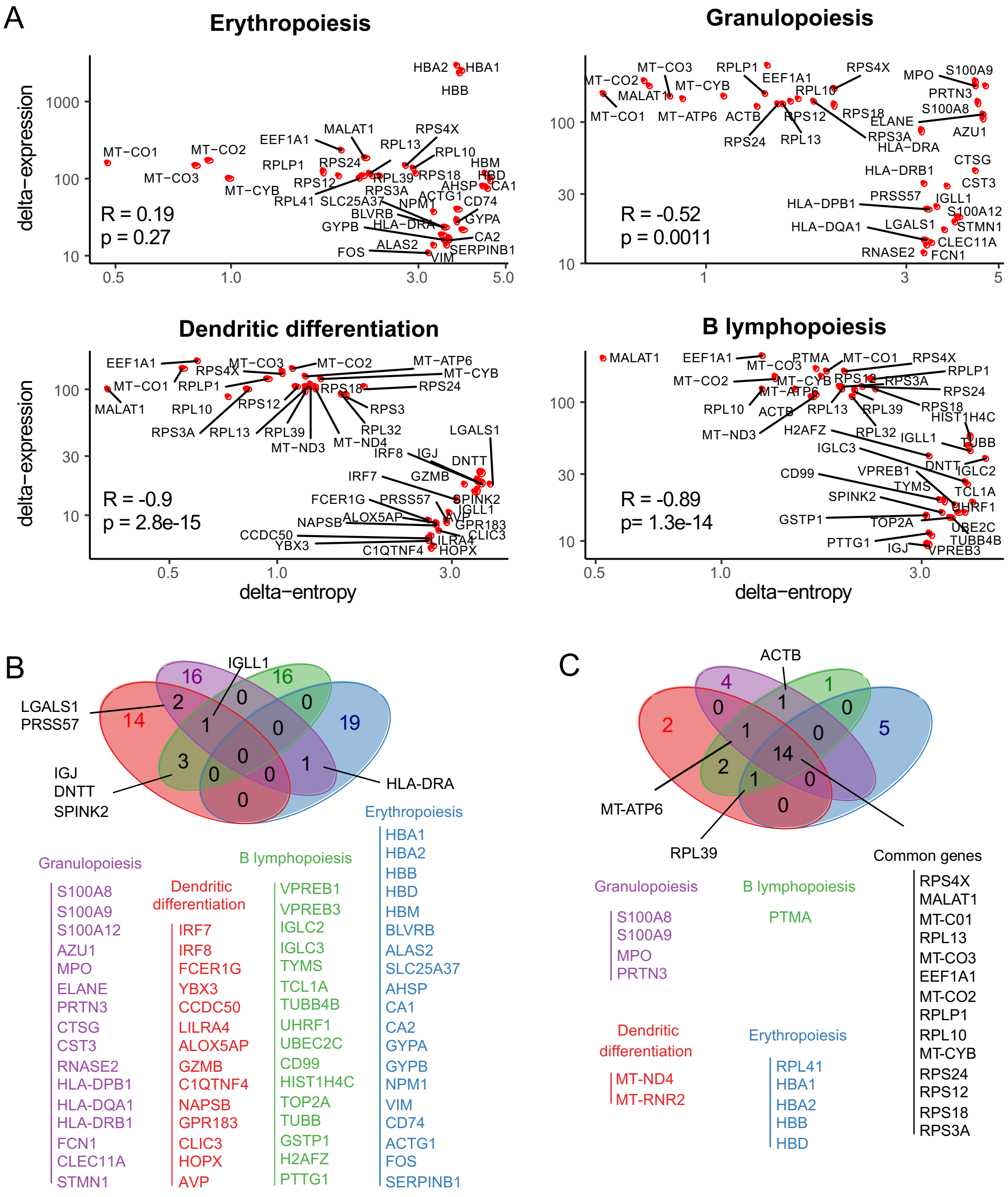

Figure S6: Most delta-entropic and most delta-expressed genes along hematopoietic differentiation (HBM1).

(A) For each gene (red dots on the graphs), delta-expression is represented as a function of delta-entropy (logarithmic scale), in the 4 different hematopoietic differentiation pathways. **B-C)** Venn diagram of the 20 most delta entropic **(B)** and 20 most delta expressed **(C)** genes during the different hematopoietic differentiation pathways.

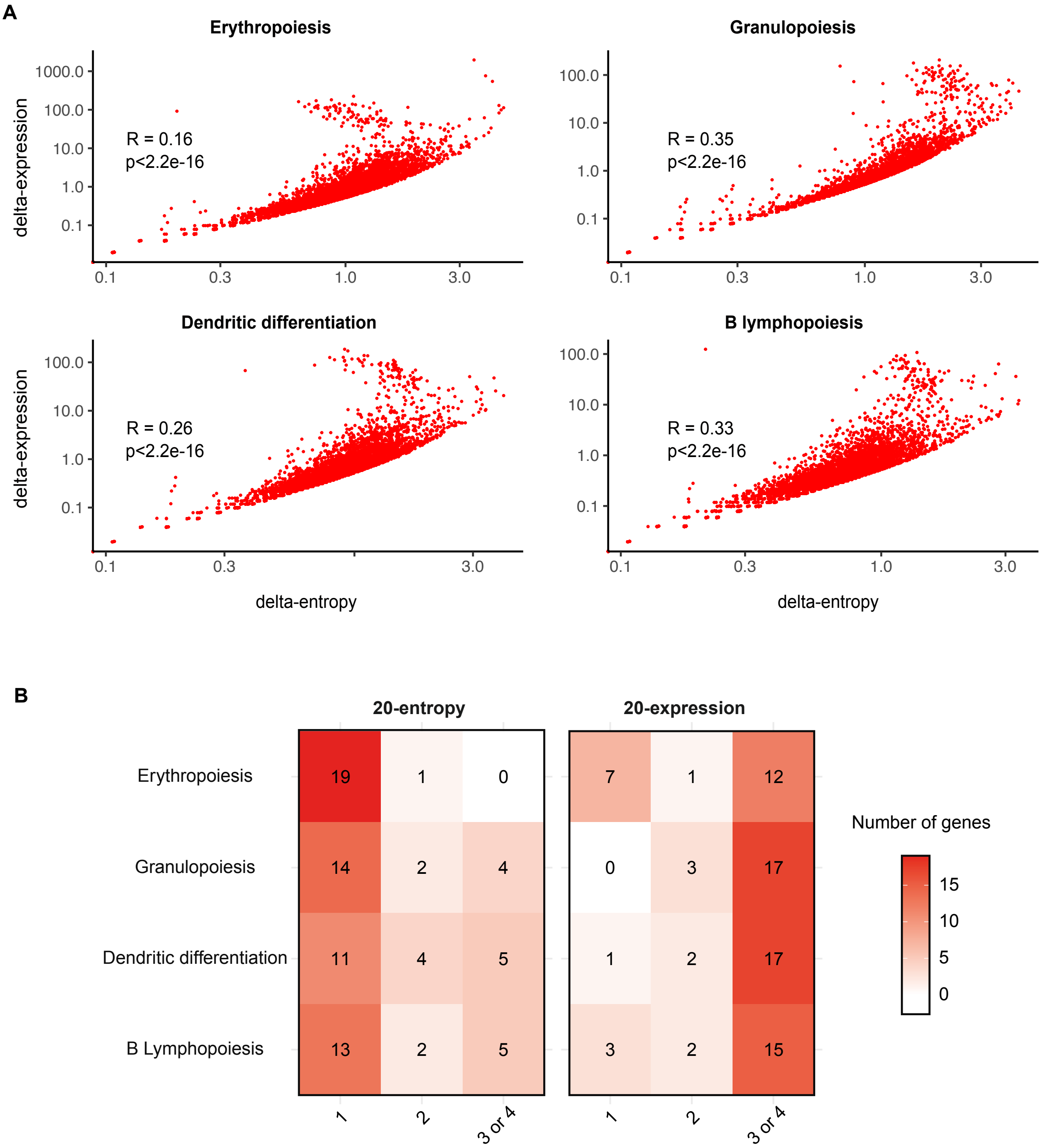

Figure S7: delta-entropic and delta-expressed genes along hematopoietic differentiation (HBM2).

A) For each gene (red dots on the graphs), delta-expression is represented as a function of delta-entropy (logarithmic scale), in the 4 different hematopoietic differentiation pathways. **B)** Overlay between the different lists. Among the 20 genes that are the most delta-entropic within the erythropoietic pathway, only 1 was also appearing in the most delta-entropic in another differentiation pathway. On the contrary, among the 20 genes with the highest delta-expression in the granulopoiesis pathway, 17 were also appearing in the 20-expression lists in at least two other differentiation pathways.

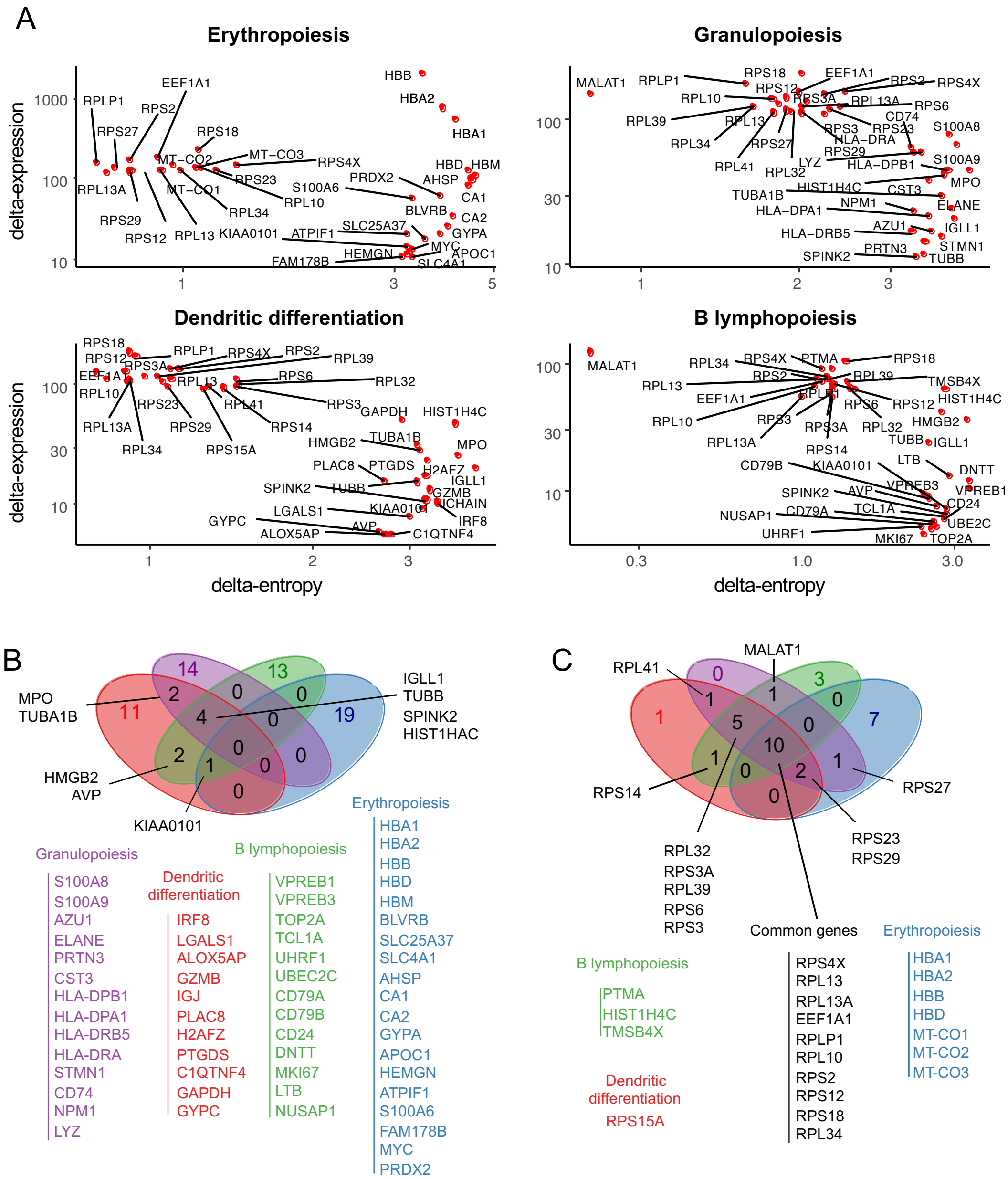

Figure S8: Most delta-entropic and most delta-expressed genes along hematopoietic differentiation (HBM2).

(A) For each gene (red dots on the graphs), delta-expression is represented as a function of delta-entropy (logarithmic scale), in the 4 different hematopoietic differentiation pathways. **B-C)** Venn diagram of the 20 most delta entropic **(B)** and 20 most delta expressed **(C)** genes during the different hematopoietic differentiation pathways.

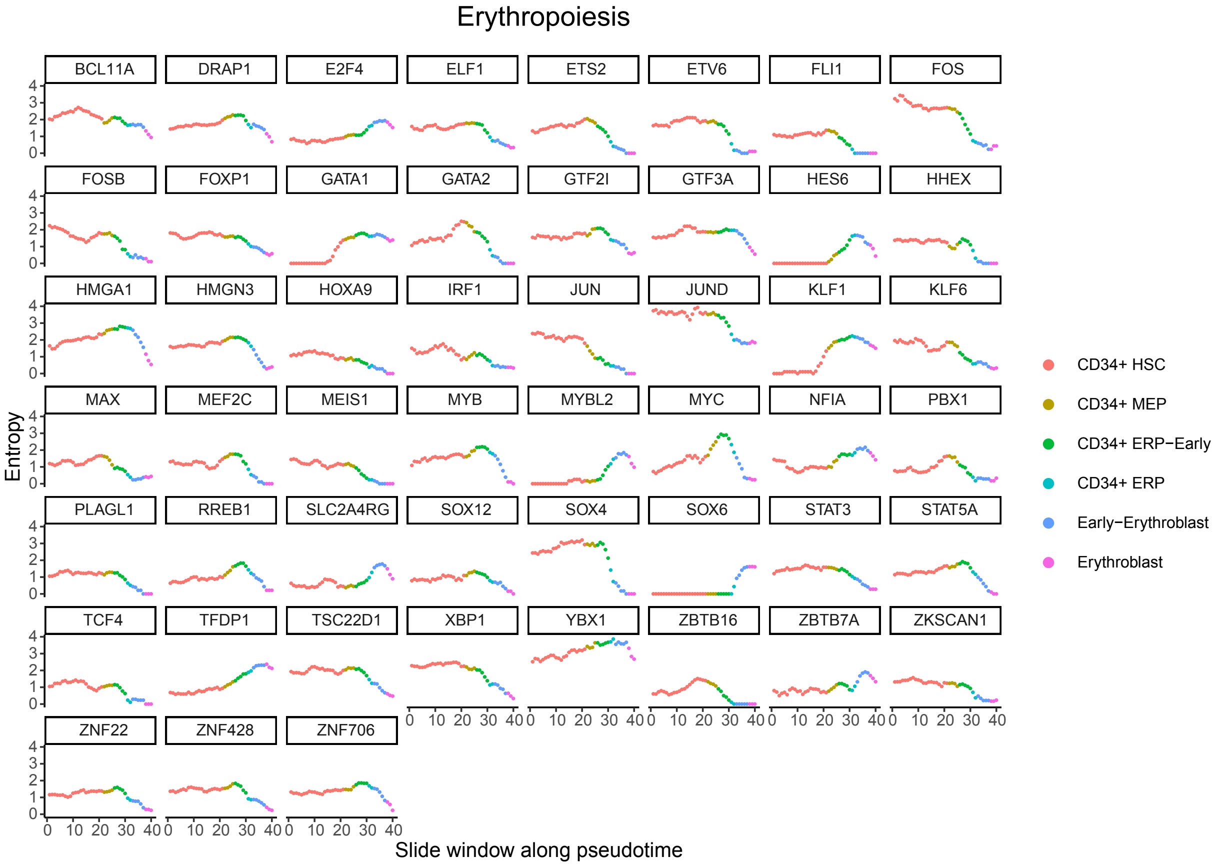

Figure S9: Cell-to-cell gene expression variability of transcription factors belonging to the 1000 most delta entropic genes during Erythropoiesis (HBM1).

Cell populations belonging to erythropoiesis were first selected and then ordered according to the pseudotime calculated by Slingshot. The intercellular entropy of each transcription factor was then calculated on a sliding window of 50 cells which moves across the pseudotime with a step of 10 cells (the color of each point on the graph correspond to the nature of the first cell in the corresponding sliding window).

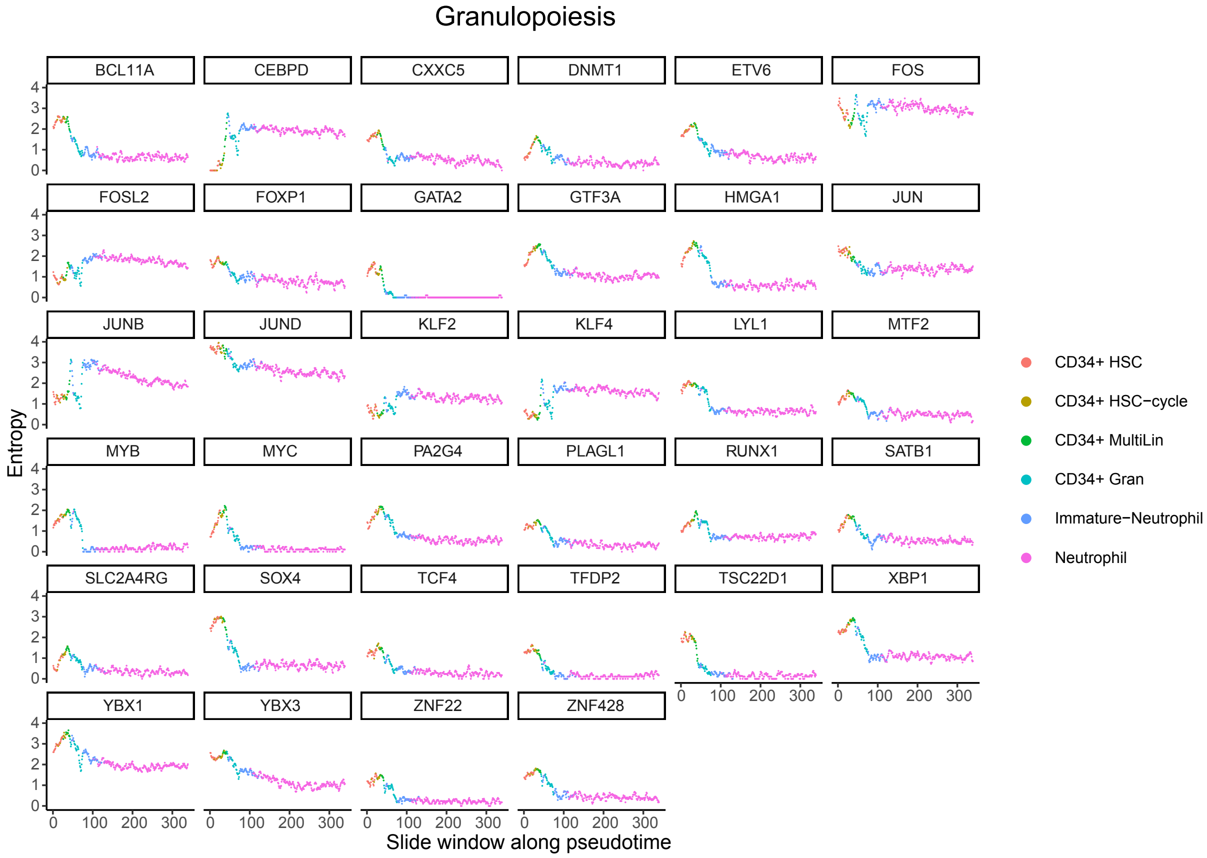

Figure S10: Cell-to-cell gene expression variability of transcription factors belonging to the 1000 most delta entropic genes during Granulopoiesis (HBM1).

Cell populations belonging to granulopoiesis were first selected and then ordered according to the pseudotime calculated by Slingshot. The intercellular entropy of each transcription factor was then calculated on a sliding window of 50 cells which moves across the pseudotime with a step of 10 cells (the color of each point on the graph correspond to the nature of the first cell in the corresponding sliding window).

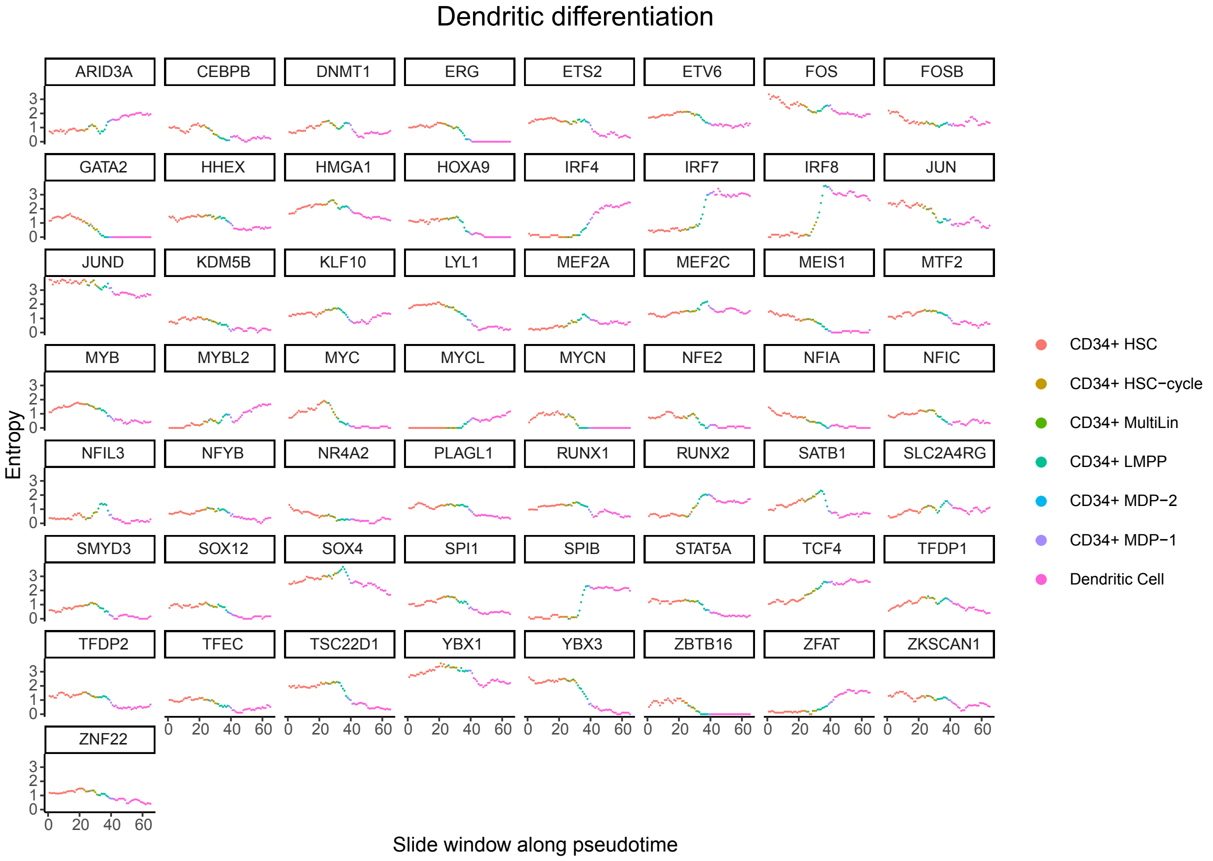

Figure S11: Cell-to-cell gene expression variability of transcription factors belonging to the 1000 most delta entropic genes during dendritic differentiation (HBM1).

Cell populations belonging to dendritic differentiation were first selected and then ordered according to the pseudotime calculated by Slingshot. The intercellular entropy of each transcription factor was then calculated on a sliding window of 50 cells which moves across the pseudotime with a step of 10 cells (the color of each point on the graph correspond to the nature of the first cell in the corresponding sliding window).

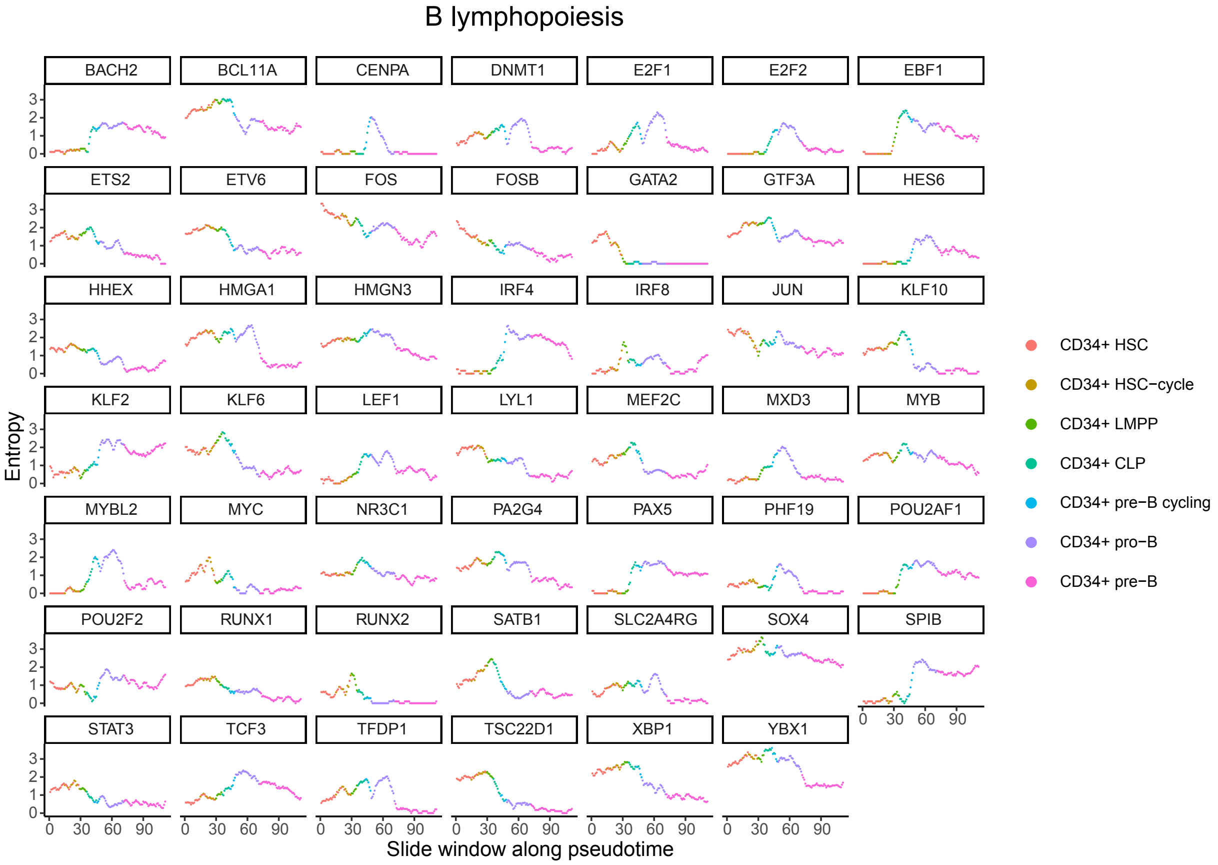

Figure S12: Cell-to-cell gene expression variability of transcription factors belonging to the 1000 most delta entropic genes during B lymphopoiesis (HBM1).
Cell populations belonging to B lymphopoieses were first selected and then ordered according to the pseudotime calculated by Slingshot. The intercellular entropy of each transcription factor was then calculated on a sliding window of 50 cells which moves across the pseudotime with a step of 10 cells (the color of each point on the graph correspond to the nature of the first cell in the corresponding sliding window).

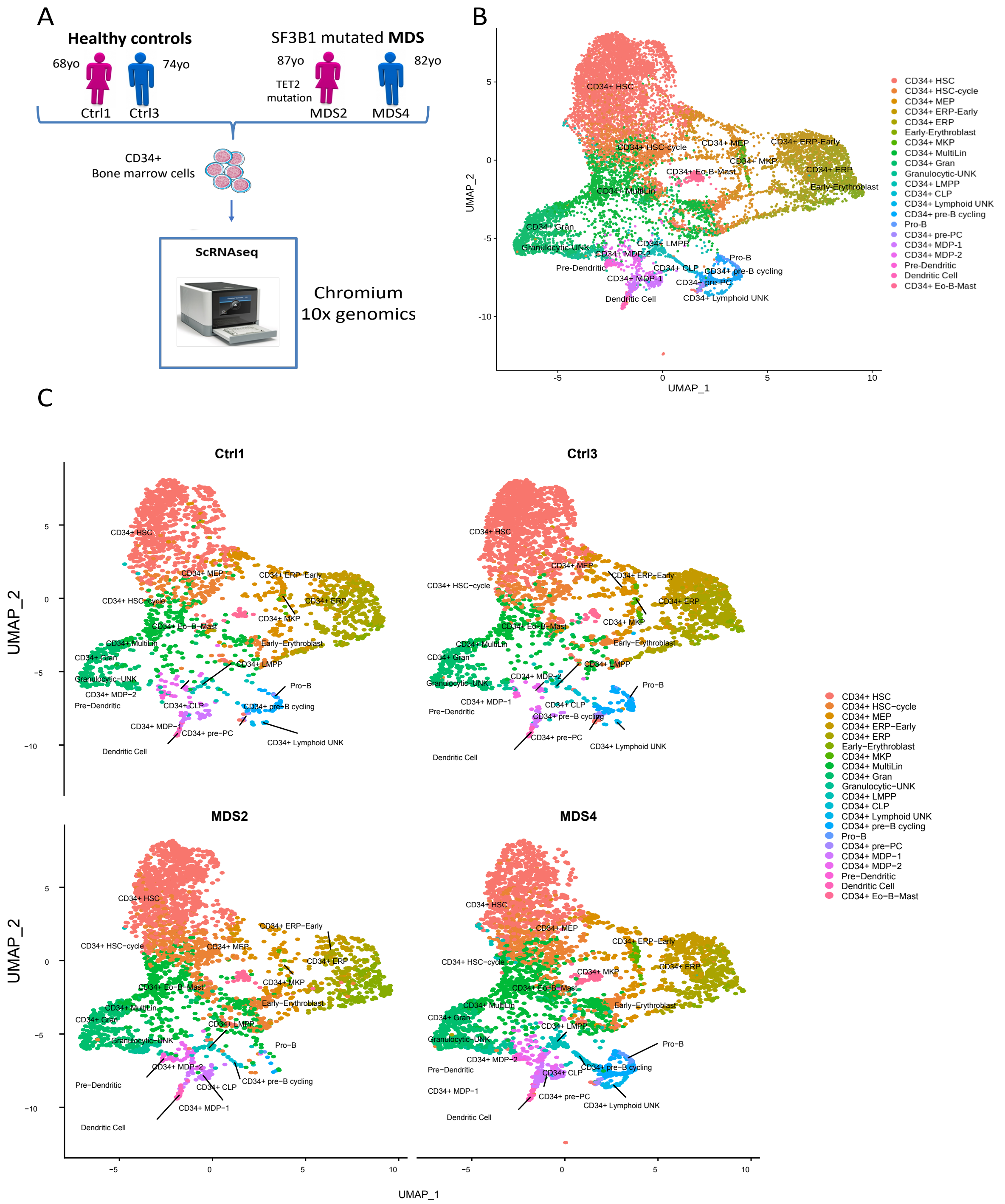

Figure S13: Transcriptional landscape of the HSPC compartment of SF3B1 mutated MDS and healthy elderly subjects.

A) Outline of experimental approach. CD34+ HSPCs were isolated from bone marrow of healthy elderly controls (Ctrl1, Ctrl3) and SF3B1 mutated. scRNAseq was performed using chromium 10x genomics technology. **B-C)** Analysis of 10x Genomics scRNAseq data from 12689 cells, combining the 4 samples. The cells annotated by SingleR are classified into 21 different subtypes, each represented by a different color. **B)** UMAP of HSPC landscape combining the 4 samples **C)** UMAP of HSPC landscape from each individual

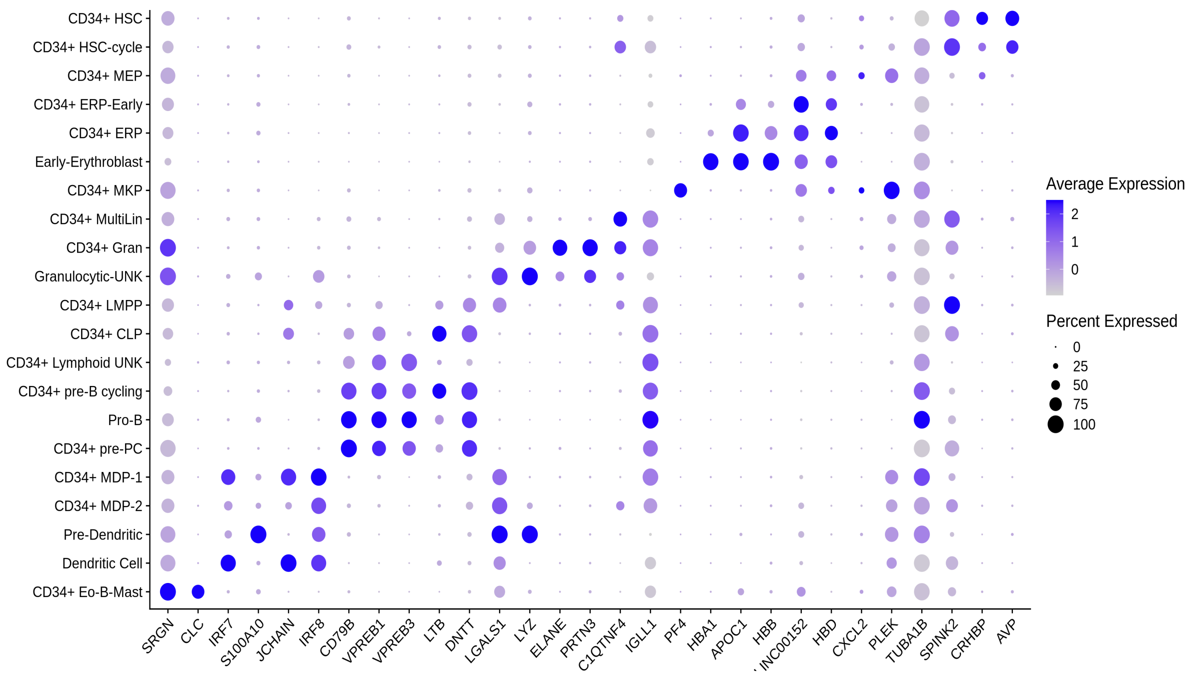

Figure S14: Expression values of selected marker genes for all cell sub-populations of the HSPC compartment of SF3B1 mutated MDS and healthy elderly subjects.

Circle color shows mean scaled expression values and circle size represents the proportion of expressing cells per sub-populations.

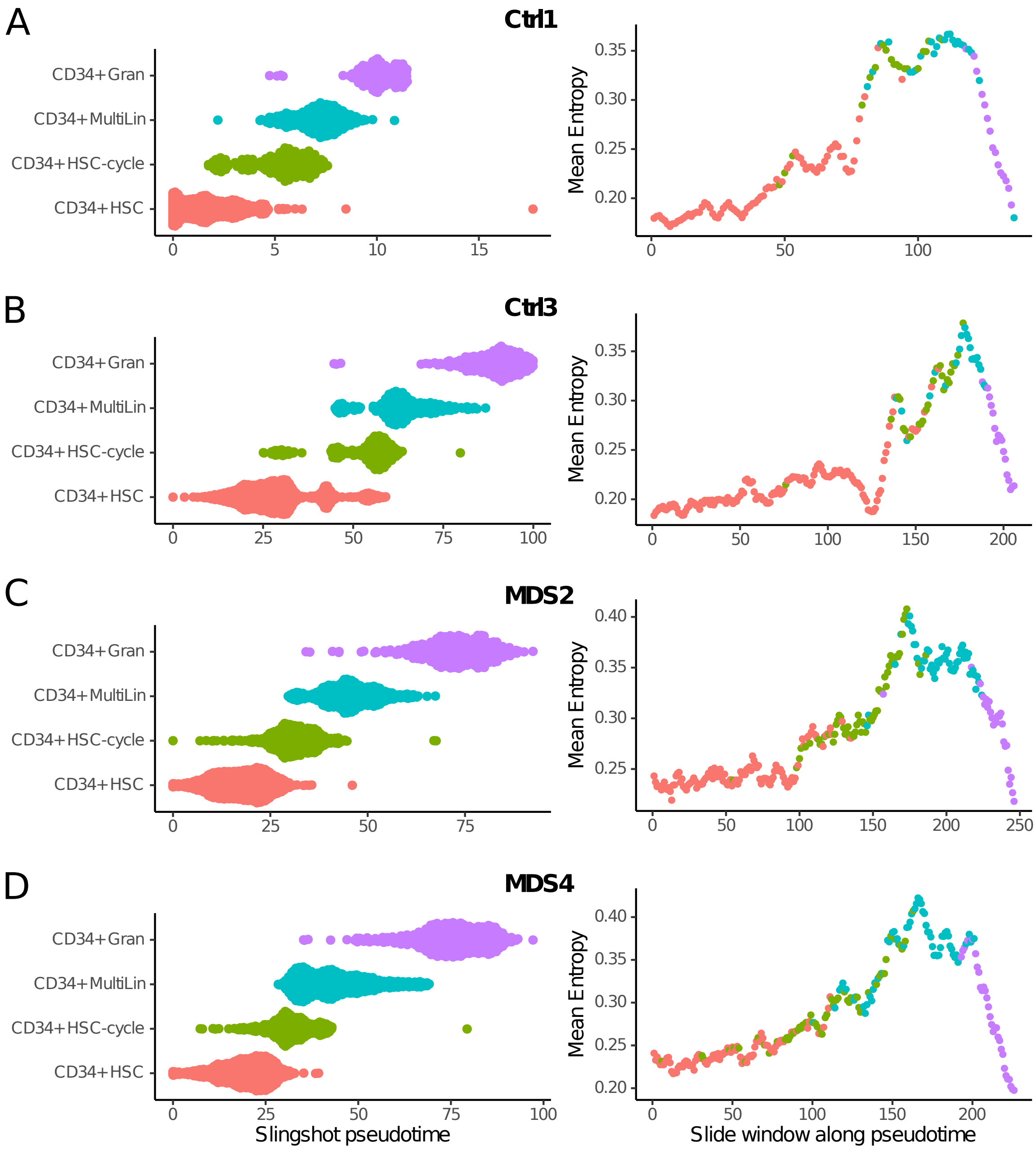

Figure S15: Evolution of cell-to-cell gene expression variability during granulopoiesis in elderly subjects and SF3B1-mutated MDS.

For each sample individually, cell populations belonging to granulopoiesis were first selected and then ordered according to the pseudotime calculated by Slingshot. The average intercellular entropy of all genes was then calculated on a sliding window of 50 cells which moves across the pseudotime with a step of 10 cells (the color of each point on the graph correspond to the nature of the first cell in the corresponding sliding window). **A)** Ctrl1 **B)** Ctrl3 **C)** MDS2 **D)** MDS4

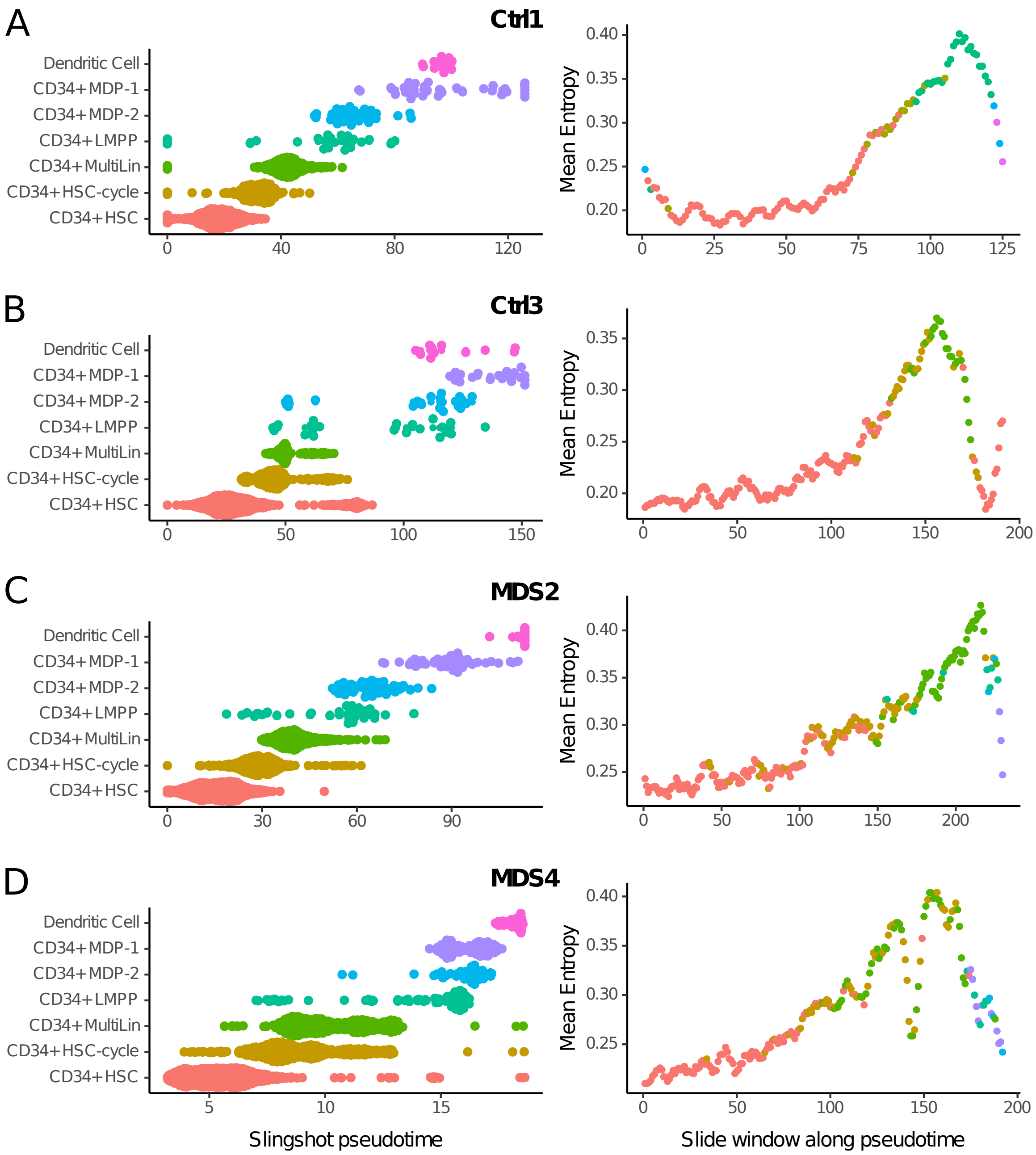

Figure S16: Evolution of cell-to-cell gene expression variability during dendritic differentiation in elderly subjects and SF3B1-mutated MDS.

For each sample individually, cell populations belonging to dendritic differentiation were first selected and then ordered according to the pseudotime calculated by Slingshot. The average intercellular entropy of all genes was then calculated on a sliding window of 50 cells which moves across the pseudotime with a step of 10 cells (the color of each point on the graph correspond to the nature of the first cell in the corresponding sliding window). **A)** Ctrl1 **B)** Ctrl3 **C)** MDS2 **D)** MDS4

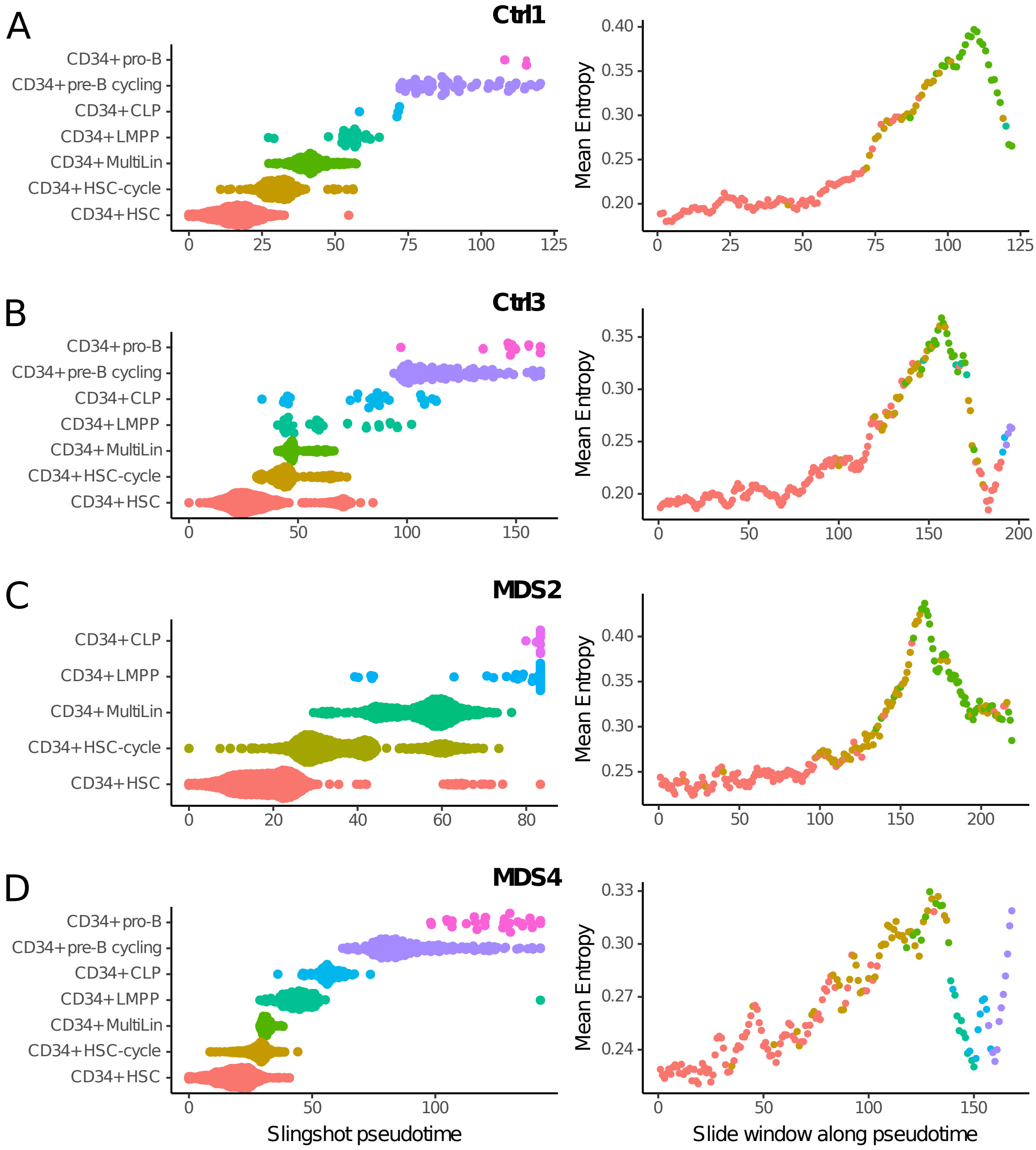

Figure S17: Evolution of cell-to-cell gene expression variability during B lymphopoiesis in elderly subjects and SF3B1-mutated MDS.

For each sample individually, cell populations belonging to B lymphopoiesis were first selected and then ordered according to the pseudotime calculated by Slingshot. The average intercellular entropy of all genes was then calculated on a sliding window of 50 cells which moves across the pseudotime with a step of 10 cells (the color of each point on the graph correspond to the nature of the first cell in the corresponding sliding window). **A)** Ctrl1 **B)** Ctrl3 **C)** MDS2 **D)** MDS4

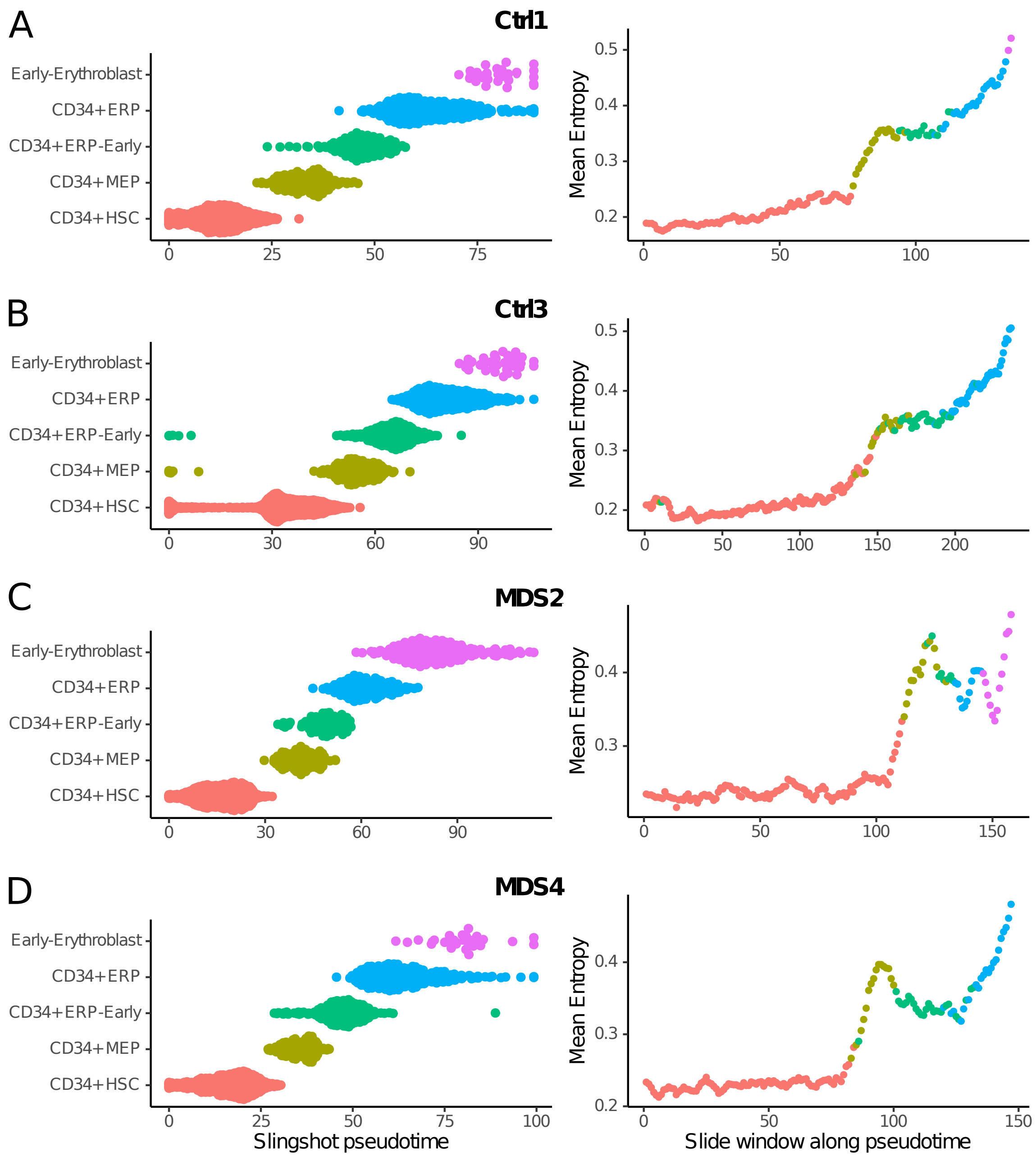

Figure S18: Evolution of cell-to-cell gene expression variability during Erythropoiesis in elderly subjects and SF3B1-mutated MDS.

For each sample individually, cell populations belonging to erythropoiesis were first selected and then ordered according to the pseudotime calculated by Slingshot. The average intercellular entropy of all genes was then calculated on a sliding window of 50 cells which moves across the pseudotime with a step of 10 cells (the color of each point on the graph correspond to the nature of the first cell in the corresponding sliding window). **A)** Ctrl1 **B)** Ctrl3 **C)** MDS2 **D)** MDS4

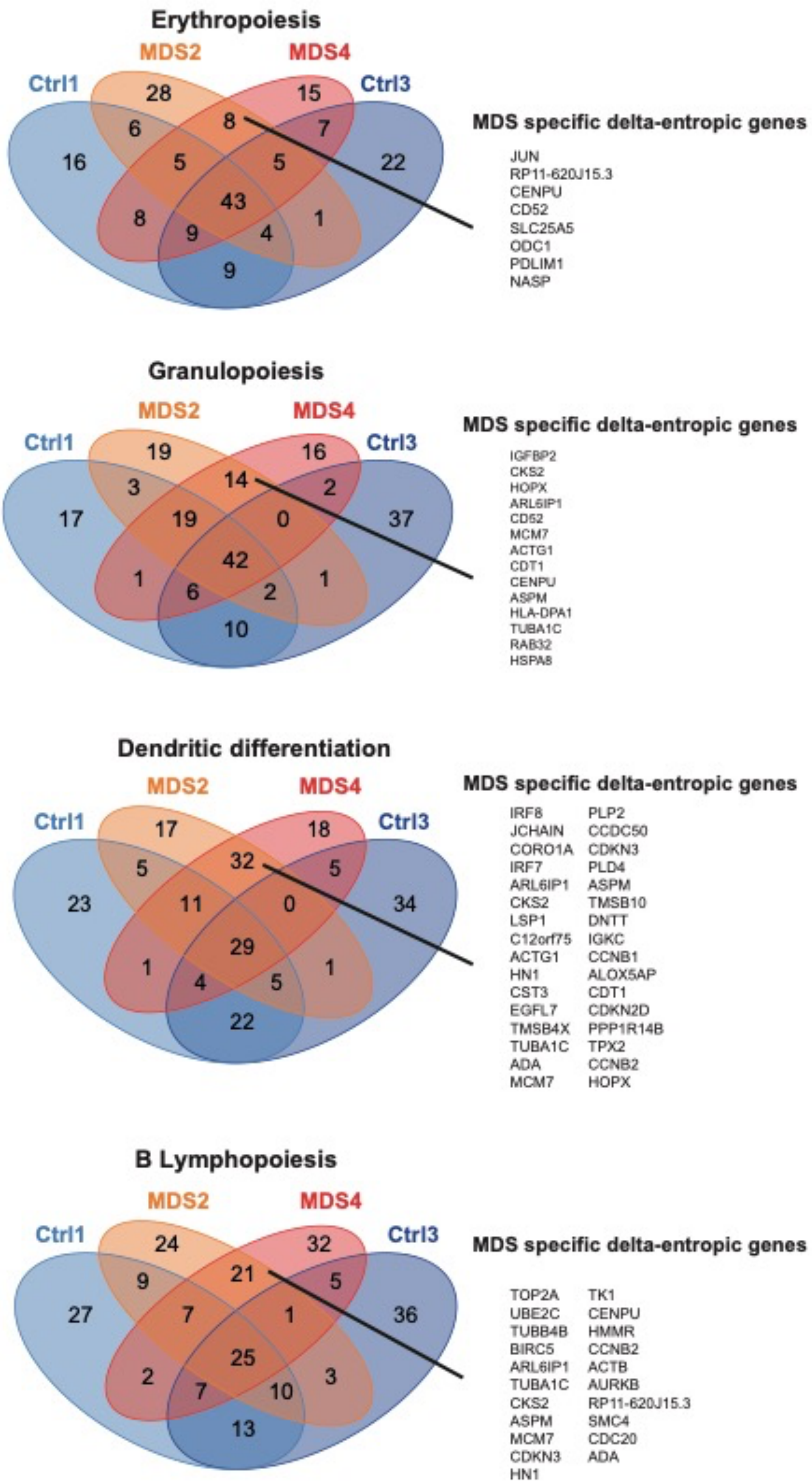
Erythropoiesis
MDS2
MDS4
Ctrl1
Ctrl3
28
15
6
8
7
16
5
5
22
43
8
9
4
1
9
MDS specific delta-entropic genes
JUN
RP11-620J15.3
CENPU
CD52
SLC25A5
ODC1
PDLIM1
NASP
Granulopoiesis
MDS2
MDS4
Ctrl1
Ctrl3
19
16
3
14
2
17
19
0
37
42
1
6
2
1
10
MDS specific delta-entropic genes
IGFBP2
CKS2
HOPX
ARL6IP1
CD52
MCM7
ACTG1
CDT1
CENPU
ASPM
HLA-DPA1
TUBA1C
RAB32
HSPA8
Dendritic differentiation
MDS2
MDS4
Ctrl1
Ctrl3
17
18
5
32
5
23
11
0
34
29
1
4
5
1
22
MDS specific delta-entropic genes
IRF8
JCHAIN
CORO1A
IRF7
ARL6IP1
CKS2
LSP1
C12orf75
ACTG1
HN1
CST3
EGFL7
TMSB4X
TUBA1C
ADA
MCM7
PLP2
CCDC50
CDKN3
PLD4
ASPM
TMSB10
DNTT
IGKC
CCNB1
ALOX5AP
CDT1
CDKN2D
PPP1R14B
TPX2
CCNB2
HOPX
B Lymphopoiesis
MDS2
MDS4
Ctrl1
Ctrl3
24
32
9
21
5
27
7
1
36
25
2
7
10
3
13
MDS specific delta-entropic genes
TOP2A
UBE2C
TUBB4B
BIRC5
ARL6IP1
TUBA1C
CKS2
ASPM
MCM7
CDKN3
HN1
TK1
CENPU
HMMR
CCNB2
ACTB
AURKB
RP11-620J15.3
SMC4
CDC20
ADA

Figure S19: Comparison of the 100 most delta entropic genes along hematopoietic differentiation between MDS patients and age-matched healthy subjects.

Venn diagrams of the 100 most delta entropic genes during the different hematopoietic differentiation pathways. MDS specific delta-entropic genes are specified for each hematopoietic differentiation pathway.

HBM1

Erythropoiesis	CD34+ HSC	CD34+ MEP	CD34+ ERP-Early	CD34+ ERP	Early-Erythroblast	Erythroblast
# cells	204	39	45	23	54	79

Granulopoiesis	CD34+ HSC	CD34+ HSC-cycle	CD34+ MultiLin	CD34+ Gran	Immature-Neutrophil	Neutrophil
# cells	209	99	117	277	416	2322

Dendritic differentiation	CD34+ HSC	CD34+ HSC-cycle	CD34+ MultiLin	CD34+ LMPP	CD34+ MDP-2	CD34+ MDP-1	Dendritic Cell
# cells	198	98	109	62	12	29	289

B Lymphopoiesis	CD34+ HSC	CD34+ HSC-cycle	CD34+ MultiLin	CD34+ LMPP	CD34+ CLP	CD34+ pre-B cycling	CD34+ pro-B	CD34+ Pre-B
# cells	205	98	116	63	72	29	289	455

HBM2

Erythropoiesis	CD34+ HSC	CD34+ MEP	CD34+ ERP-Early	CD34+ ERP	Early-Erythroblast	Erythroblast
# cells	163	69	72	100	180	74

Granulopoiesis	CD34+ HSC	CD34+ HSC-cycle	CD34+ MultiLin	CD34+ Gran	Immature-Neutrophil	Neutrophil
# cells	164	114	140	102	2273	2956

Dendritic Differentiation	CD34+ HSC	CD34+ HSC-cycle	CD34+ MultiLin	CD34+ LMPP	CD34+ MDP-2	CD34+ MDP-1	Dendritic Cell
# cells	164	114	140	42	37	56	272

B Lymphopoiesis	CD34+ HSC	CD34+ HSC-cycle	CD34+ MultiLin	CD34+ LMPP	CD34+ CLP	CD34+ pre-B cycling	CD34+ pro-B	CD34+ Pre-B
# cells	164	106	23	42	40	23	62	40

Supplementary table 1: Distribution of cellular subpopulations for each differentiation pathway in HBM1 and HBM2 dataset.

Patient	Ctrl1	Ctrl3	MDS2	MDS4
Age	68	74	87	82
Sex	F	M	F	M
Diagnosis	Healthy ctrl	Healthy ctrl	MDS-RS	MDS-RS
R-IPSS	NA	NA	low	very low
Hb, g/dL	14.3	15.5	10	10,6
Platelets, G/L	220	190	342	287
Neutrophils, G/L	1.97	3	1.4	3,46
Monocytes, G/L	0.47	0.46	0.1	0,96
Lymphocytes, G/L	1.77	1.63	0.5	1,74
Bone marrow cellularity	NA	NA	High	Very high
Erythroid precursors, %	NA	NA	60	52
Myeloid precursors, %	NA	NA	30	43
Megacaryocytes	NA	NA	Presents	Numerous
Bone marrow blasts, %	NA	NA	2	1
Ring sideroblasts, %	NA	NA	25	50
Karyotype	NA	NA	46,XX,del(11)(q14][3] /46,sl,?del(20)(q12)[2] /46,X,del(X)(q21),add(5)(q?31), del(11)(q14),add(17)(q2?2)[12] /46,XX[2]	46, XY [20]
Somatic mutations (VAF%)	Not detected	Not detected	TET2, p.Q769X (44%) SF3B1, p.K700E (43%)	SF3B1, p.K666RK (35%)

Supplementary table 2: Clinico-biological features of patients from whom HSPCs were harvested for the scRNA-seq experiment.

Erythropoiesis

Sample	CD34+ HSC	CD34+ MEP	CD34+ ERP-Early	CD34+ ERP	Early-Erythroblast
Ctrl1	754	176	156	284	24
Ctrl3	1415	194	347	423	29
MDS2	1100	126	76	143	182
MDS4	824	158	253	252	24

Granulopoiesis

Sample	CD34+ HSC	CD34+ HSC-cycle	CD34+ MultiLin	CD34+ Gran
Ctrl1	754	188	256	208
Ctrl3	1415	240	229	221
MDS2	1100	618	475	311
MDS4	825	496	601	380

Dendritic differentiation

Sample	CD34+ HSC	CD34+ HSC-cycle	CD34+ MultiLin	CD34+ LMPP	CD34+ MDP-2	CD34+ MDP-1	Dendritic Cell
Ctrl1	754	188	256	25	32	32	13
Ctrl3	1415	240	229	21	23	18	11
MDS2	1100	618	475	35	42	47	25
MDS4	825	496	601	86	79	94	38

B lymphopoiesis

Sample	CD34+ HSC	CD34+ HSC-cycle	CD34+ MultiLin	CD34+ LMPP	CD34+ CLP	CD34+ pre-B cycling	CD34+ pro-B
Ctrl1	754	188	256	25	3	39	2
Ctrl3	1415	240	229	21	18	76	9
MDS2	1100	618	475	35	8	0	0
MDS4	820	489	525	86	70	150	22

Supplementary table 3: Distribution of cellular subpopulations for each differentiation pathway in Ctrl1, Ctrl3, MDS2 and MDS4 samples.

Erythropoiesis	Granulopoiesis	Dendritic differentiation	B Lymphopoiesis
HBB	ELANE	MPO	IGLL1
BLVRB	PRTN3	IGLL1	KIAA0101
KIAA0101	AZU1	KIAA0101	LGALS1
AHSP	MPO	TUBA1B	H2AFZ
HBD	IGLL1	HMGB2	STMN1
CA1	CTSG	STMN1	DNTT
APOC1	KIAA0101	H2AFZ	HMGB2
PRDX2	TUBA1B	LGALS1	TUBA1B
HMGB2	HMGB2	C1QTNF4	C1QTNF4
PLEK	CFD	HIST1H4C	TYMS
S100A6	STMN1	TYMS	TUBB
TUBB	SRGN	CALR	AVP
ATPIF1	H2AFZ	TUBB	HMGN2
TYMS	LGALS1	AVP	LTB
H2AFZ	C1QTNF4	PLAC8	DUT
DUT	HIST1H4C	HMGB1	MGST1
STMN1	LYZ	ENO1	IGFBP7
FAM178B	TYMS	HMGN2	PRSS57
TUBA1B	CALR	GAPDH	HMGB1
CKS2	TUBB	PRSS57	GAPDH
HIST1H4C	AVP	FABP5	HIST1H4C
UBE2C	PLAC8	CENPF	SMIM24
SYNGR1	HMGB1	RAN	GYPC
AVP	DUT	CLEC11A	MKI67
HNRNPAB	ENO1	IGFBP7	CKS1B
TOP2A	HMGN2	SLC25A5	
HMGN2	GAPDH	MGST1	
CKS1B	HSPA5	GYPC	
SPINK2	KIAA0125	SMIM24	
MPC2	PRSS57		
CENPF	FABP5		
HMGB1	FTH1		
PTTG1	CENPF		
YBX1	RAN		
PCNA	CLEC11A		
RAN	CST7		
TUBB4B	IGFBP7		
REXO2	RNASE2		
NCL	MGST1		
LMNA	YBX1		
KLF1	GYPC		
TFRC	ACTB		
HBA1			

Supplementary table 4: Common delta-entropic genes between MDS patients and age-matched healthy subjects during the different hematopoietic differentiation pathways.
